# COVID-19 prediction using Caviar Squirrel Jellyfish Search Optimization technique in fog-cloud based architecture

**DOI:** 10.1371/journal.pone.0295599

**Published:** 2023-12-21

**Authors:** Shanthi Amgothu, Srinivas Koppu

**Affiliations:** School of Computer Science Engineering and Information Systems, Vellore, India; TU Wien: Technische Universitat Wien, AUSTRIA

## Abstract

In the pandemic of COVID-19 patients approach to the hospital for prescription, yet due to extreme line up the patient gets treatment after waiting for more than one hour. Generally, wearable devices directly measure the preliminary data of the patient stored in capturing mode. In order to store the data, the hospitals require large storage devices that make the progression of data more complex. To bridge this gap, a potent scheme is established for COVID-19 prediction based fog-cloud named Caviar Squirrel Jellyfish Search Optimization (CSJSO). Here, CSJSO is the amalgamation of CAViar Squirrel Search Algorithm (CSSA) and Jellyfish Search Optimization (JSO), where CSSA is blended by the Conditional Autoregressive Value-at-Risk (CAViar) and Squirrel Search Algorithm (SSA). This architecture comprises the healthcare IoT sensor layer, fog layer and cloud layer. In the healthcare IoT sensor layer, the routing process with the collection of patient health condition data is carried out. On the other hand, in the fog layer COVID-19 detection is performed by employing a Deep Neuro Fuzzy Network (DNFN) trained by the proposed Remora Namib Beetle JSO (RNBJSO). Here, RNBJSO is the combination of Namib Beetle Optimization (NBO), Remora Optimization Algorithm (ROA) and Jellyfish Search optimization (JSO). Finally, in the cloud layer, the detection of COVID-19 employing Deep Long Short Term Memory (Deep LSTM) trained utilizing proposed CSJSO is performed. The evaluation measures utilized for CSJSO_Deep LSTM in database-1, such as Mean Squared Error (MSE) and Root Mean Squared Error (RMSE) observed 0.062 and 0.252 in confirmed cases. The measures employed in database-2 are accuracy, sensitivity and specificity achieved 0.925, 0.928 and 0.925 in K-set.

## 1. Introduction

The industry of medical care with the new innovation has widespread importance in the day-to-day life of humans. Nevertheless, the essential challenges experienced by medical care organizations are to collect accurate information and provide an extreme nature of organizations in the present atmosphere. In recent years, the pandemic coronavirus 2019 (COVID-19) has been alleged all over the world as a fundamental issue caused by viral contamination, which directly affects the various organs of our human body. In spite of that, a supreme managing and controlling scheme should be introduced to detect COVID-19 specifically in the areas which are highly affected with a high range of spread. All over the world, this kind of disease is the prime test for the agencies of healthcare to manage in real-time. Smart sensors can be embedded at any region with the enhanced development of the innovation of the Internet of Things (IoT). The enhanced growth of innovation and the impending unavoidable technological revolt produce possible effectual applications based on healthcare [1]. COVID-19 is the prime pandemic diagnosis where an enormous amount of dullness and death occurred all over the world. The early stage of this diagnosis shows fever, cough, headache, lack of smell and taste with unpredicted oxygen saturation [2,3]. According to the investigations, 40% to 60% of COVID-19 cases are mysterious which is a momentous restraint for the industry of medical service. The infection fails to recognize and screen the disease accurately by the preceding and old developments [4].

Fog and cloud computing development as well as embedded IoT schemes have been elected as the fundamental one due to energy handling, more capacity limit and intuition of effectual and accurate information [4]. The trio-logical computing module is related to the medical care scheme, which is employed for the examination of disaster, enhancing sound living at minimal outlay and viewing the far site that is unreachable, then only the safety measures are taken in the real world. In spite of the points of interest in the developed scheme, data mining progression for data assessment and evaluation enhances the quality of medical care organization by authorized valuable chances. The novel model of IoT fog-cloud computing has been effectually enhanced by several industries in terms of medical to achieve some errands like managing medical care and the outcomes of transportation with unpredictable delays of time. In the fog layer, the fog hubs accumulate the standard information from several sensors and IoT-based devices connected with some pre-arranged devices to handle constant information and assign the outcomes to the user in the real world [4]. COVID-19 has spread around 215 countries with enormous amount of cases and deaths. Throughout this eruption, each part of our everyday life has been intensely affected. One of the prime difficulties is its physical transmission rate in the course of droplet inhalation or contact with tainted areas. Recent investigations have established that asymptomatic patients are specifically infectious, since human beings are inclined to stay away from others by showing clear symptoms, but asymptomatic humans cannot be quickly identified [5].

Early recognition of infected cases and the sensible allotment of partial medical resources are important [5,6]. A COVID-19 symptom includes sore throat, running nose and cough. The virus may aggravate people’s death with weak immune systems. The spread of this disease is transmitted through physical contact. Normally, healthy people may be infected by the contact of breath and mucous contact of the affected person [7]. Some symptoms may be linked with chest X-ray (CXR) and it may be employed to treat this disease. A CXR may be employed as a visual indicator of COVID-19 by the radiologists who led to the establishment of an enormous amount of deep learning (DL) techniques and its examination may reveal that the detection correctness of COVID-19 infected patients employing chest radiography images [8]. Convolutional neural networks (CNNs) obtained existing assessments in the medical image field provided by sufficient data. Such assessment is achieved by training on labeled data and fine-tuning an enormous amount of factors. CNNs can effortlessly over fit on minimal databases since it has a large amount of parameters; thus, the efficacy of generalization is relative to the dimension of the labeled data. With an adequate amount and diversity of samples, the prime confront in the domain of medical imaging is minimal databases. The collection of medical images is an extremely luxurious and tedious progression that needs the participation of radiologists and investigators. Also, due to the recent outbreak of COVID-19 outbreak, adequate data on CXR images is complex to congregate [8].

The role of this examination is to establish a model for COVID-19 in fog-cloud named CSJSO_Deep LSTM. Firstly, the healthcare IoT sensor layer is employed to gather information about the patient’s condition and then the routing progression is conducted by proposed CSJSO. Secondly, the fog layer is utilized to detect COVID-19 by DNFN which is trained employing RNBJSO. Lastly, the cloud layer is to predict COVID-19 by employing Deep LSTM, which is trained by CSJSO.

Proposed CSJSO_Deep LSTM for COVID-19 prediction: A potent framework CSJSO_Deep LSTM is introduced for COVID-19 prediction in the fog-cloud model. Here, COVID-19 is detected in the fog layer by utilizing DNFN, which is trained by RNBJSO. Here, RNBJSO is the incorporation of NBO and ROA. COVID-19 is accomplished in the cloud layer by Deep LSTM which is trained by CSJSO. Here, CSJSO is the combination of CSSA and JSO, where CSSA is formed by CAViar and SSA.

The remainder segment is as follows: In fragment 2, the prior techniques of COVID-19 prediction and detection are described. The system module of the fog-cloud model is designed and decrypted in segment 3. Probe 4 enumerates the proposed model with the three layers and its training algorithm. Lastly, the outcomes of the model are deliberated in segment 5 with the future scope.

## 2. Motivation

In recent times, deep learning strategies are trending to detect COVID-19 in the course of radiological images. So, the investigators are motivated to develop a scheme for detecting the COVID-19 disease based on the fog-cloud model by learning the prior models as well as by experiencing the benefits and drawbacks of those models.

### 2.1 Literature survey

Wang B, et al. [5] developed Reinforcement Learning Approach. This scheme was helpful in the early recognition of COVID-19 cases and also for governments and decision-making establishments. However, it did not utilize more data to validate and amend this early recognition model at escalated accurateness. Waheed A, et al. [8] introduced the Auxiliary Classifier GAN. This method was enhanced to detect COVID-19 and it attains robust structure. Nevertheless, it failed to improve the x of synthetic chest image quality by training a progressive growing GAN. Togacar, M., et al. [7] devised Deep learning (DL) models. This scheme obtained the entire rate of classification and effectually contributed diagnose of COVID-19. Nevertheless, it did not implement several structuring models to improve the databases. Ozturk, T., et al. [9] established deep neural networks (DNN). DNN was utilized to aid radiologists in authorizing their original screening, and also it was used through the cloud to screen the patients directly. But, this method can only utilize the limited amount of data that failed to obtain this model more robust.

Fan, D.P., et al. [10] created Lung Infection Segmentation Deep Network (Inf-Net). This strategy enhanced the learning capability and attained with supreme assessment. This method had a slight variance in accuracy while assuming the non-infected slices. By implementing an additional slice-wise classifier for electing the affected slice was an effectual solution for neglecting the assessment the drop on non-infected slices. Muller, D., et al. [1] designed U-Net architecture. This method produced high latent to be included as a medical decision system for COVID-19 quantitative performance and disease examination in a medical environment and it showed that the medical image segmentation pipeline was capable of training precise and robust techniques devoid of over fitting on limited data. However, the prior database with minimal data resulted as partial and imprecise labels. Ranjbarzadeh, R., et al. [11] generated Cascade Convolutional neural network (CNN). This scheme outperformed more categorization efficacy by means of stability and time consumption. The drawback of this module was that the pulmonary nodules inside the lung adjacent to the border of a lesion were not accurately detected from the affected tissue. Liu, J., et al. [12] devised a transfer learning (TL) framework. This technique obtained supreme segmentation precision and surpassed cutting-edge schemes both quantitatively and qualitatively and also it deliberated the efficaciousness of the two-stage TL model, the generalization of this scheme, and the efficacy of modules.

### 2.2 Challenges

The difficulties experienced by prior models in terms of COVID-19 prediction in the fog-cloud model are elucidated below.

➢ An approach DNN devised in [7] was well performed in the categorization of COVID-19 data. However, DL-based evaluation was not performed employing data images of other organs infected by the virus from the point of a COVID-19 specialist.
➢ Even though in [9], the method was capable of conducting binary and multi-class tasks, it failed to be employed in remote areas in countries infected by COVID-19 to conquer a lack of radiologists.
➢ Although Inf-Net in [10] accomplished superior achievements in segmenting infected regions, it failed to generate an end-to-end framework in order to obtain multi-class infection labeling.
➢ In [1], the devised model surpassed the prior semantic segmentation models for lungs and COVID-19-affected areas. However, this model failed to implement the semantic segmentation of COVID-19 in medical diagnosis for examining the assessment and its robustness.
➢ Recently, the COVID-19 prediction in the fog-cloud model deduced the death rate by the healthcare sector. However, the security protocol is not applied to achieve the extreme assessment by employing the fog-cloud model.

## 3. System model

**Fig 1:**
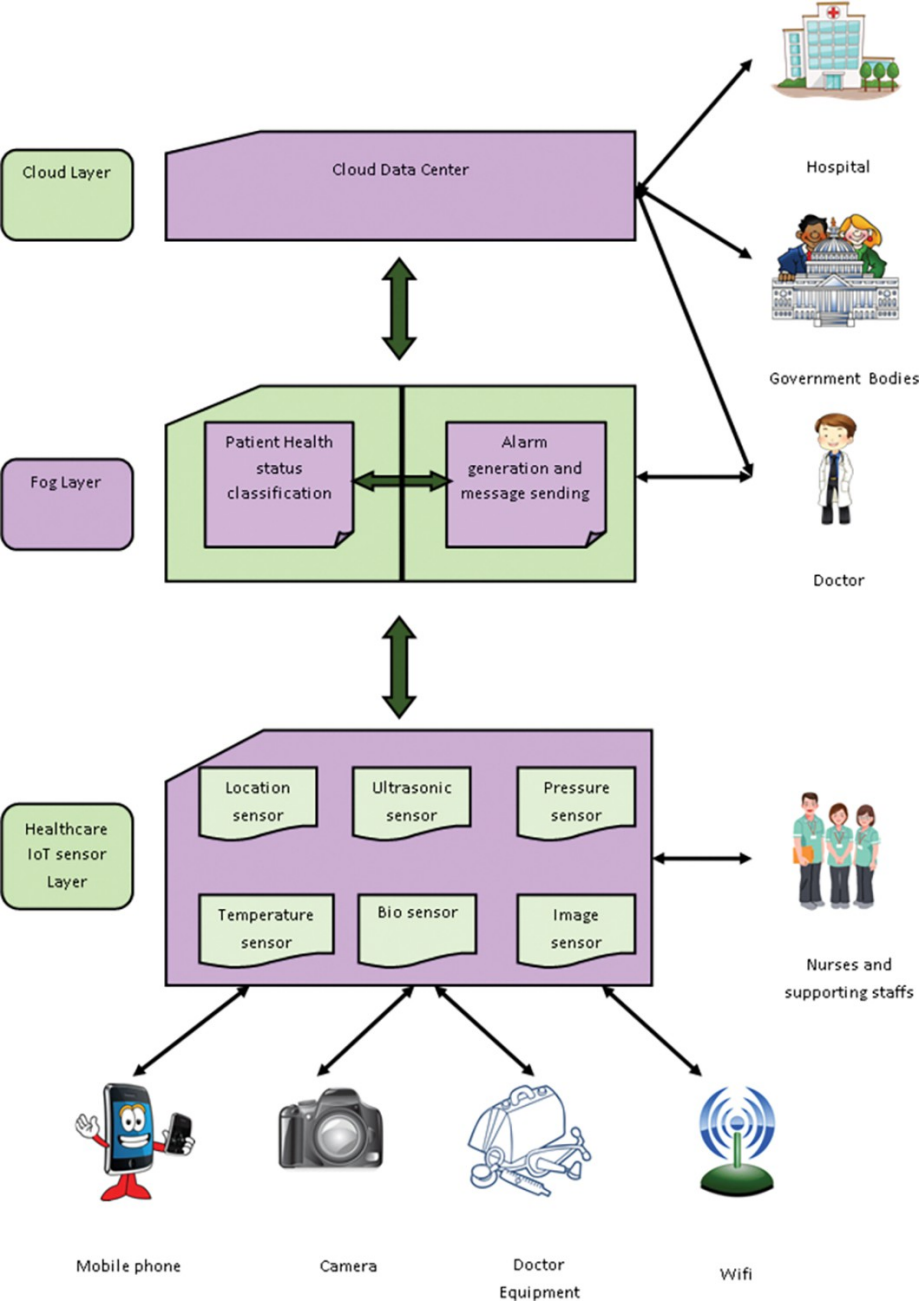
Systematic model of fog-cloud COVID prediction.

This system model [13] comprises a healthcare IoT sensor layer, fog layer and cloud layer. Firstly, in the healthcare IoT sensor layer, the IoT sensor layer will gather the details relevant to the patient’s health conditions by employing location sensors, ultrasonic sensors, pressure sensors, temperature sensors, biosensors and image sensors. Secondly, the fog layer is positioned amid the cloud layer and healthcare IoT sensor layer. The doctors are linked directly to the fog layer and they will respond to the queries. In order to reduce the delay, the data should be transferred from the fog layer to the cloud layer as well as the energy consumed by devices employed at the fog layer is extremely minimal. If the delay and time response are high, it will be difficult to respond and treat the patients as well as it leads the patients to a dead state. So, the fog layer is employed to overcome this challenge. Lastly, the cloud layer accumulates the details at the cloud data center. Fig 1 represents the system model.

## 4. Proposed CSJSO_Deep LSTM for COVID-19 prediction in fog layer

The proposed architecture comprises three layers, like Healthcare IoT sensor layer, Fog layer, and cloud layer. The proposed system of three layers and its training algorithm are briefly elaborated in beneath sub-fragments.

### 4.1 Healthcare IoT sensor layer

At the Healthcare IoT sensor layer, the nodes collect Computed Tomography (CT) images from the patients and the routing process is carried out by employing the proposed CSJSO. Here, CSJSO is devised by integrating the CSSA and JSO [14], where CSSA is obtained by CAViar [15] and SSA [16] with the help of fitness parameters like, energy, link lifetime, distance, and trust.

#### 4.1.1 Routing

Routing is employed to elect the optimal path in the network then only data packets are transferred from sender to receiver in a safe manner. This process improves the significance of network-based services. The optimal path selection is performed in terms of objective functions like energy, link lifetime (LLT), distance and trust.

#### 4.1.2 Training algorithm of routing using proposed CSJSO

SSA [16] is inspired from the behaviour of squirrels and it is a vigorous way to conduct the locomotion called gliding. Normally, this model is about food searching and it acquires large-scale optimal solutions with improved convergence distinctiveness. In CAViar [15], is based on the distribution of returns in terms of quantile features. Moreover, the imprecise factors may be examined by quantile process with time employing the process of autoregressive and regression quantile model. In JSO [14], the algorithm is motivated by the movement of jellyfish present in the ocean which are searching for food. By the amalgamation of SSA, CAViar and JSO, the proposed model CSJSO obtained the well performance with more efficaciousness. The training algorithm of routing employed presented model CSJSO is elucidated in the below sub-sections.

a) Position encoding

**Fig 2:**
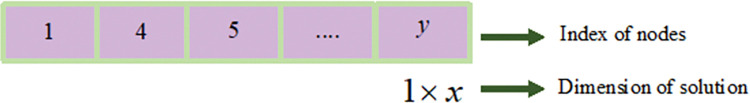
Solution encoding.

The solution encoding is employed to recognize the optimal solution in a given search space for COVID-19 prediction. The size of the solution is 1×*x* and the index of nodes as *y* in the range of 1≤*v*≤*y*, which is described in Fig 2.

b) Fitness Measure

It is employed to analyze the utmost solution and minimal value is elected as the optimal path that is illustrated as,

$$N=\frac{e_{z}+x_{zy}+(1-h_{zy})+{ℑ}_{zy}}{4}$$ (1)

Here, *e*_*z*_ is the energy consumption, *h*_*zy*_ is the Euclidean distance, ℑ_*zy*_ is the trust, and *x*_*zy*_ is the LLT.

**Step1: Initialization**

This phase is employed to initialize the population with random solutions of flying squirrels that are illustrated by,

$$K_{l}=K_{LB}+UB(0,1)\times (K_{UB}-K_{LB})$$ (2)

where, *K*_*LB*_,*K*_*UB*_ represents the upper and lower bound and *UB*(0,1) indicates the random number that is uniformly distributed in the interval of (0,1).

**Step 2: Compute fitness measure**

It is employed to identify an optimal solution with the greatest resultant for every squirrel employing Eq (1).

**Step 3: Evaluate the position**

The position of squirrel is arranged from the minimal order using the values of fitness. Hence, the squirrel with minimal value is transferred to hickory nut tree and the exploration deeds are exaggerated due to the presence of predators. The natural deeds are altered by the possibility of an upgrade position.

**Step 4: Upgrade the new solution**

The squirrels are transferred to the forest to find food through their non-existence of predators. Nevertheless, it migrates to the nearby direction to hide from the predator and thus the foraging deeds of squirrels are arithmetically formulated in three cases.

***Case-1*:** The squirrels in acorn nut trees migrate to hickory nut tree, and the new position is given by,

$$K_{uv}^{v+1}=K_{uv}^{v}+h_{e}Z_{o}\times (K_{iv}^{v}-K_{uv}^{v})$$ (3)

From CAViaR [17], the upgrade expression is formulated as,

$$Y_{pq}^{q}=\delta_{0}+\sum_{i=1}^{x}\delta_{i}Y_{pq}^{q-i}+\sum_{j=1}^{y}z(Y_{pq}^{q-i})$$ (4)

$$K_{uv}^{v+1}=K_{iv}^{v}(1-h_{e}\times Z_{o})+h_{e}\times Z_{o}\times (\gamma_{0}+\gamma_{1}(K_{uv}^{v-1}+k(K_{uv}^{v-1}))+\gamma_{2}(K_{uv}^{v-2}+k(K_{uv}^{v-2})))$$ (5)

Subtracting $K_{uv}^{v+1}$on both sides,

$$K_{uv}^{v+1}-K_{uv}^{v}=K_{iv}^{v}(1-h_{e}\times Z_{o})+h_{e}\times Z_{o}\times (\gamma_{0}+\gamma_{1}(K_{uv}^{v-1}+k(K_{uv}^{v-1}))+\gamma_{2}(K_{uv}^{v-2}+k(K_{uv}^{v-2})))-K_{uv}^{v}$$ (6)

The upgrade expression from JSO is employed to enhance the better performance and it is illustrated by,

$$A_{p}(q+1)=A_{p}(q)+rand(0,1)\cdot A^{*}-\alpha rand(0,1)\times \lambda$$ (7)

Assume,

$$A_{p}(q+1)=K_{uv}^{v+1}$$ (8)

$$A_{p}(q)=K_{uv}^{v}$$ (9)

$$A^{*}=K_{best}^{v}$$ (10)

Substituting Eqs (8), (9) and (10) in Eq (7),

$$K_{uv}^{v}=K_{uv}^{v+1}-rand(0,1)\cdot K_{best}^{v}+\alpha \;rand(0,1)\times \lambda$$ (11)

Substituting Eq (11) in Eq (6),

$$\begin{array}{l} K_{uv}^{v+1}=K_{iv}^{v}(1-h_{e}\times Z_{o})+h_{e}\times Z_{o}\times (\gamma_{0}+\gamma_{1}(K_{uv}^{v-1}+k(K_{uv}^{v-1}))+\gamma_{2}(K_{uv}^{v-2}+k(K_{uv}^{v-2})))\\ \;-K_{uv}^{v+1}+rand(0,1)\cdot K_{best}^{v}-\alpha \;rand(0,1)\times \lambda +K_{uv}^{v} \end{array}$$ (12)

$$\begin{array}{l} 2K_{uv}^{v+1}=K_{iv}^{v}(1-h_{e}\times Z_{o})+h_{e}\times Z_{o}\times (\gamma_{0}+\gamma_{1}(K_{uv}^{v-1}+k(K_{uv}^{v-1}))+\gamma_{2}(K_{uv}^{v-2}+k(K_{uv}^{v-2})))\\ \;+rand(0,1)\cdot K_{best}^{v}+\alpha \;rand(0,1)\times \lambda +K_{uv}^{v} \end{array}$$ (13)

$$K_{uv}^{v+1}=\frac{\begin{array}{l} K_{iv}^{v}(1-h_{e}\times Z_{o})+h_{e}\times Z_{o}\times (\gamma_{0}+\gamma_{1}(K_{uv}^{v-1}+k(K_{uv}^{v-1}))+\gamma_{2}(K_{uv}^{v-2}+k(K_{uv}^{v-2})))\\ \;+rand(0,1)\cdot K_{best}^{v}+\alpha \;rand(0,1)\times \lambda +K_{uv}^{v} \end{array}}{2}$$ (14)

Here, γ implies the p-vector of unknown parameters, $K_{uv}^{v-1},K_{uv}^{v-2}$ is indicated as the flying squirrel on across nut tree at *v*−1 and *v*−3, *k*(*K*) is the fitness of flying squirrel, random gliding distance is represented as *h*_*e*_, where *Z*_*o*_ = 1.9, $K_{iv}^{v}$ is the flying squirrel on hickory nut tree, γ = 3, and the mean location is signified as λ.

If the above expression is not satisfied, then the random location *R*_1_ will be chosen for further process.

***Case-2*:** In normal tress, the squirrels migrate towards the acorn nut tree to finish their daily necessities that is computed by,

$$K_{mv}^{v+1}=K_{mv}^{v}+h_{e}Z_{o}\times (K_{uv}^{v}-K_{mv}^{v})$$ (15)

Else, the random location *R*_2_ will be elected.

***Case-3*:** In acorn nut trees, the squirrels try to migrate to the hickory nut tree, which is determined by,

$$K_{mv}^{v+1}=K_{mv}^{v}+h_{e}Z_{o}\times (K_{iv}^{v}-K_{mv}^{v})$$ (16)

Else, the random location *R*_3_ will be elected.

**Step 5:** Examine the seasonal constant

The seasonal constant is computed by the following expression,

$$Z_{o}^{v}=\sqrt{\sum_{n=1}^{h}{\left(K_{uv,n}^{v}-K_{iv,n}\right)}^{2}}$$ (17)

**Step 6:** Random relocation of the season

The squirrels may not discover the forest for supreme food in winter yet they live in new directions. The relocation of the squirrels is computed by,

$$K_{mq}^{new}=K_{LB}+Levy(m)+(K_{UB}-K_{LB})$$ (18)

**Step 7**: Termination

The aforesaid progress of CSJSO will be frequent till it achieves a superior solution. The pseudo-code of CSJSO is mentioned in algorithm 1.

Algorithm 1. Pseudo code of developed CSJSO.

SL. NO Pseudo code of developed CSJSO

1 Input: *K*_*l*_

2 Output: $K_{uv}^{v+1}$

3 Begin

4 Initialization population

5 Determine fitness by Eq (1)

6 Sort the location

7 While (the termination condition is not satisfied)

8 for *v* = 1 *to h*_1_; *h*_1_-flying squirrels on acorn trees

9  if *R*_1_≥*υ*-*υ* is considered as the presence of predator

10   Update the new solution by Eq (14)

11 Else

12 $K_{uv}^{v+1}$ = random location of search space

13 End

14 End

15 for *v* = 1 *to h*_2_; *h*_2_-flying squirrels on normal trees

16  if (*R*_2_≥*υ*)

17 Update the new solution by Eq (14)

18 Else

19 $K_{uv}^{v+1}$ = random location

20 End

21 End

22 for *v* = 1 *to h*_3_

23  if (*R*_3_≥*υ*)

24   Determine the shifting process of squirrels by Eq (16)

25 Else

26 $K_{uv}^{v+1}$ = random location

27 End

28 End

29 Examine seasonal constant

30 if (*G*^*v*^<*G*_*min*_)

31  Evaluate the lesser value of a seasonal constant by (18)

32 End

33  The position of squirrel on hickory nut tress is the end solution

34 End

35 Return

36 Terminate

### 4.2 Fog layer

**Fig 3:**
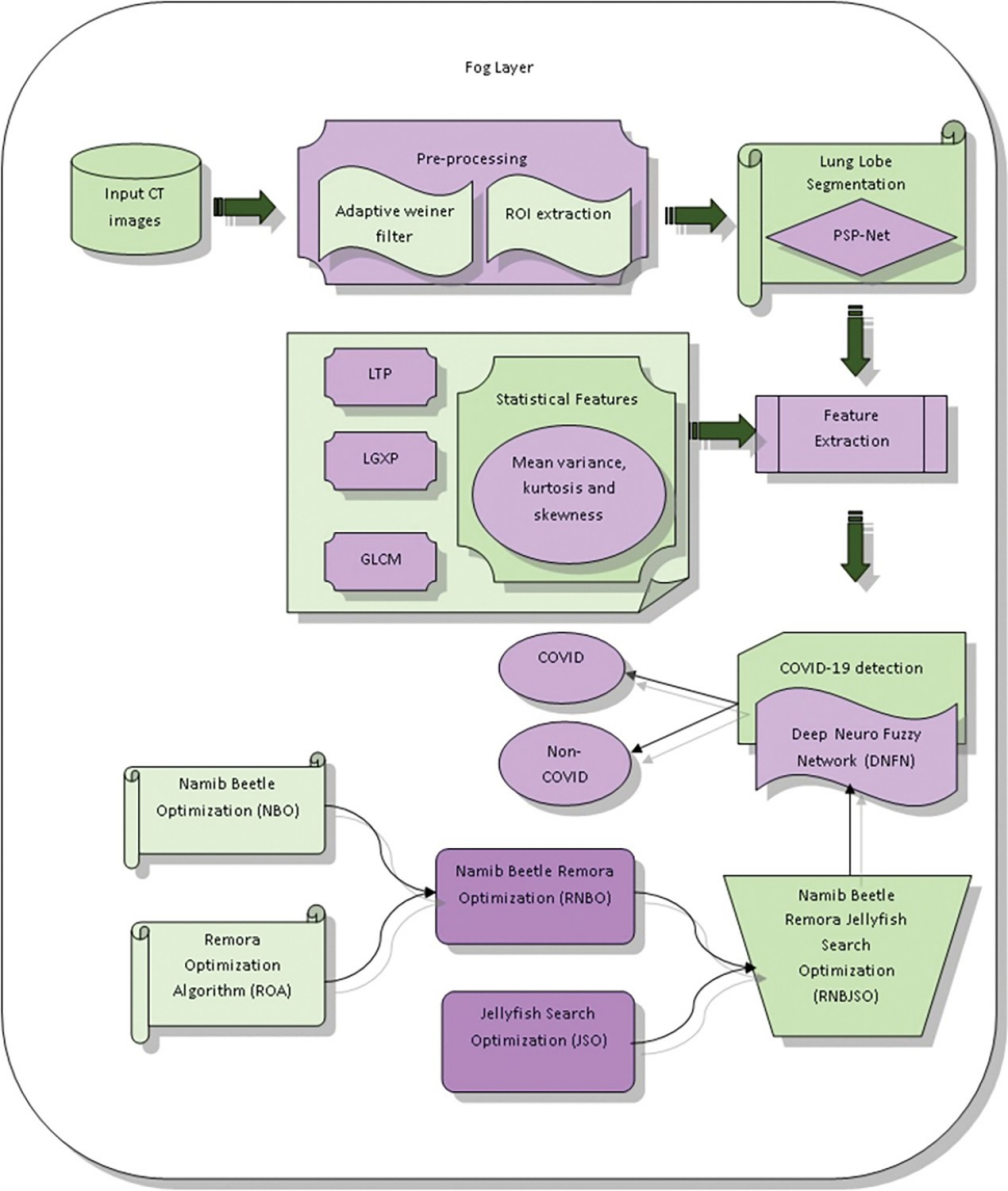
Schematic view of fog layer.

In the phase of the fog layer, the COVID patients are predicted by initializing the input CT image acquired from the database [18]. Initially, the input images are subjected to the pre-processing phase, where the noise and artifacts present in the image are neglected by employing an adaptive wiener filter [19] and ROI extraction [20]. After that, the pre-processed image is forwarded to the lung lobe segmentation in order to deduce the complexity of the image that is conducted by utilizing the Pyramid Scene Parsing Network (PSPNet) [21]. Then, the segmented image is given to the feature extraction, where the suitable features are extracted. Here, the extracted features are Local Ternary Pattern (LTP) [22], Gray Level Co-occurrence Matrix (GLCM) features [23], Local Gabor XoR Pattern (LGXP) [24], statistical features such as mean, variance, standard deviation, kurtosis, and skewness. Moreover, the extracted features are then given to the detection unit, where the COVID detection is categorized into COVID and non-COVID. The detection of COVID is done by employing a Deep Neuro Fuzzy Network (DNFN) [25], which is trained by the proposed Namib Beetle Remora JSO (RNBJSO). Here, NBRJSO is concatenated by Namib Beetle Optimization (NBO) [26] and Remora Optimization Algorithm (ROA) [27]. Fig 3 designs the illustration of the fog layer.

#### 4.2.1 Image acquisition

Considering a standard database *E* with *b* number of CT images that is computed by,

$$E=\{E_{1},E_{2},\ldots E_{x},\ldots .E_{y}\}$$ (19)

where, *E*_*a*_ is the input for the entire process occurs in the fog layer.

#### 4.2.2 Image pre-processing

The image *E*_*x*_ is forwarded to the pre-processing unit to abolish the noise and artifacts by adaptive wiener filtering as well as the extraction of ROI.

**a) Adaptive wiener filtering**

On the basis of the local variance of the image, adaptive wiener filtering [19] amends the resultant of the filter. The primary aim of this filter is to deduce the MSE among the recovered image and the actual image. This filter is extremely functional to preserve the edges and the image’s high-frequency areas.

Assume, the image filters corrupted in terms of signal intent noise is determined by,

δ(yy,zz)=ρ(yy,zz)+σ(yy,zz) (20)

where, noisy measurement denotes δ(*yy*,*zz*), noise-free image as ρ(*yy*,*zz*) and additive noise as σ(*yy*,*zz*). The purpose of abolishing noise is δ(*yy*,*zz*).

The mean and variance of pixels in various dimensions of windows for a pixel in the image is like (3+2*yy*)^2^+*yy* = 0,1,2,3 are compared, thus the window is employed by the final processing window. The smaller window filter is employed in the concise portion and the larger window filter is employed in the even portion which may improve the value and hold the texture areas and edges. It is illustrated by,

J(yy,zz)=ϖ+(1−xx+∇)*(gg(yy,zz)−ϖ) (21)

Here, the original pixel is given by *gg*(*yy*,*zz*) and *J*(*yy*,*zz*) signifies an output pixel.

**b) ROI extraction**

The image *J*(*yy*,*zz*) is given to the ROI extraction [20], where the suitable extraction may improve the chances of the medical diagnosis model accurately by detecting the significant areas in an image with COVID as it neglects the non-associated information. The outcome is symbolized as *G*_*x*_.

#### 4.2.3 Lung lobe segmentation

The *G*_*x*_ is then fed to the segmentation phase, where the lobes are individual parts of the lungs and it is the prime one to diagnose the lung disease. This phase is done by PSP-Net [21].

**a) Structure of PSP-Net**

PSP-Net [21] is the segmentation system that employs the pyramid pooling module, which comprises features in terms of four pyramid scales. The pyramid level splits the feature map to various sub-areas that appear as a pooled description for several positions that result in several dimensions. After maintaining the weight of global features, the low-size feature maps are up-sampled directly to get equivalent dimension features as actual one through the bilinear interpolation. Finally, a variety of levels of attributes are integrated as final pyramid-pooling global feature.

**Fig 4:**
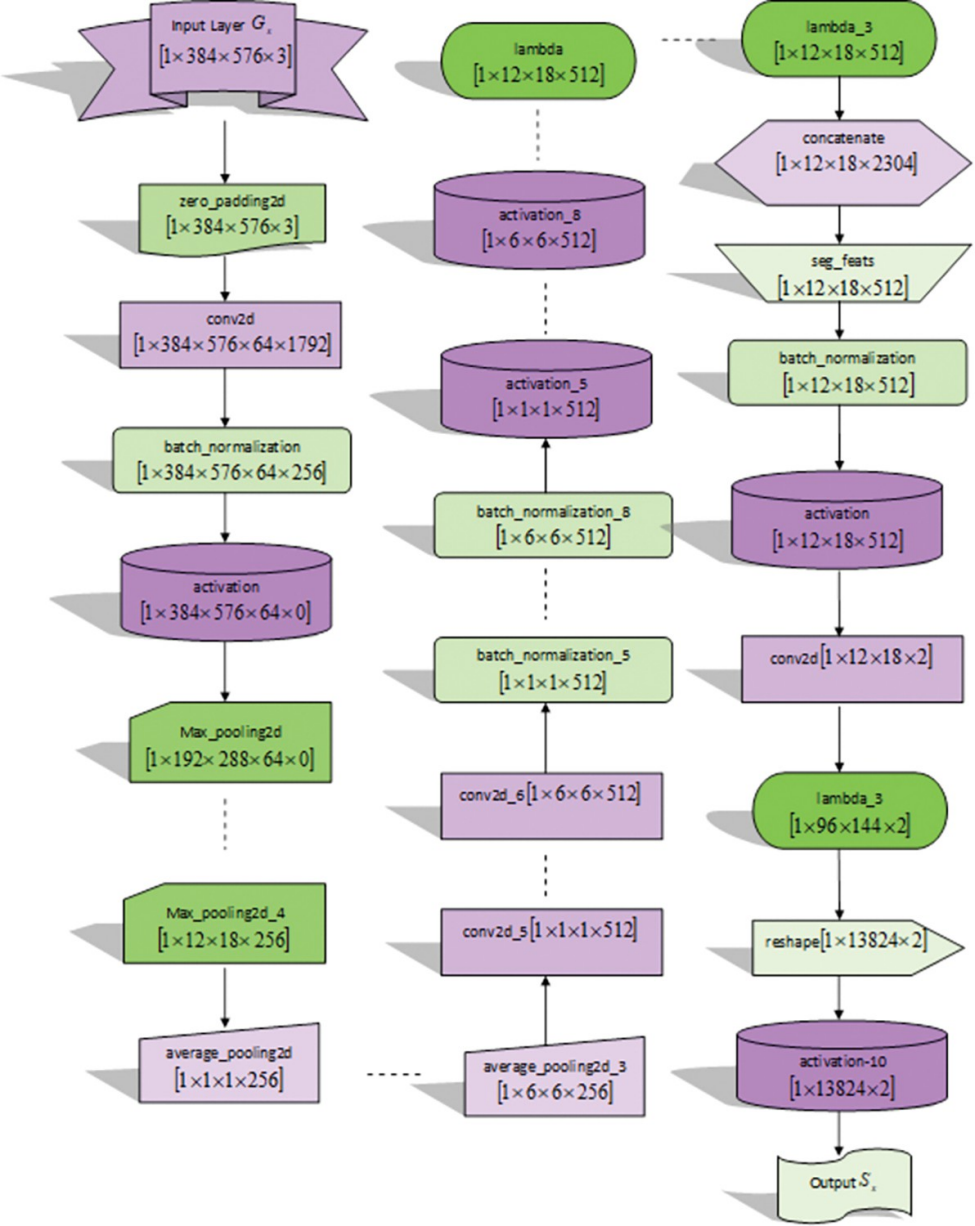
Architectural view of PSP-Net.

The numerous levels of pyramids and the dimensions of every level can be altered. They are relevant to the dimension of the feature map, which is forwarded to the pyramid pooling layer. The architecture elucidates several sub-areas by assuming various size pooling kernels in a few strides. Therefore, the multi-stage kernels should manage the reasonable gap in the illustration. The module of this pyramid pooling is a four-level with the corresponding dimensions of bin as 1×1,2×2,3×3, and 6×6. The result is illustrated by *S*_*x*_. Fig 4 depicts the architectural view of PSP-Net.

#### 4.2.4 Feature extraction

This unit is to extract the features and to obtain the suitable feature vectors and to deduce the dimensionality of the image. The segmented image *S*_*x*_ is forwarded to LTP and LGXP features to obtain suitable vectors.

*a) LTP*

It [22] comprises three valued codes like (-1, 0,1). The grey levels have a width of −ƛ to +ƛ about ν_ι_ are quantized to 0, −1 and 1. It is represented by,

$$f_{1}=d(\hbar ,\nu_{\iota },ƛ)=\{\begin{array}{l} 1,\hbar \geq \nu_{\iota }+ƛ\\ 0,|\hbar -\nu_{\iota }<ƛ\\ -1,\hbar \leq \nu_{\iota }-ƛ \end{array}$$ (22)

Here, the user-defined threshold implied as ƛ and LTP is signified as *f*_1_.

*b) LGXP*

LGXP [24] is initially classified into various ranges and then the LXP operator is employed to classify the stages of central pixel. The outcomes of the binary labels are linked uniformly as local pattern of central pixel. LGXP *f*_2_ is formulated by,

$$f_{2}=\Phi_{\theta ,l}(o_{\vartheta })={\left[\Phi_{\theta ,l}^{\Lambda },\Phi_{\theta ,l}^{\Lambda -1},\ldots ,\Phi_{\theta ,l}^{1}\right)}_{bb}={\left[\sum_{jj=1}^{\Lambda }2^{jj-1}.\Phi_{\theta ,l}^{jj}\right]}_{cc}$$ (23)

Here, *bb* and *cc* is symbolized as binary and decimal, Gabor stage map comprises scale as *l* and orientation θ implies o_ϑ_ and dimension as Λ.

Thus, the extracted features of LTP and LGXP is determined by,

$$\Psi_{m}=\{f_{1},f_{2}\}$$ (24)

*c) GLCM features*

The extracted feature Ψ_*m*_ is forwarded to GLCM features and statistical features to acquire the appropriate vectors.

GLCM [23] is a statistical model to examine the textures by considering the spatial connection of pixels. This feature regulates the traits of image texture by examining the pair of pixels with specific efficiency and the particular spatial connection repeatedly occurs in the image develops GLCM and extracts the statistical measures from the matrix.

$$f_{3}=\eta_{\beta }^{2}=\sum_{\chi =0}^{Aa-1}{\left(Xx_{\beta }(\chi )-\upsilon_{\beta }(\chi )\right)}^{2}$$ (25)

Here, the count of grey levels is *Aa*, the mean value as υ, the mean and standard deviation are implied as υ_β_, η_β_, *Xx*_β_(χ) is indicated as χ^*th*^ entry.

*d) Statistical features*

Here, the features mean, kurtosis, standard deviation, variance and skewness are deliberated in this section.

*(i)Mean*

It refers as the entire values of images categorized by the whole count of pixel values that is represented by,

$$f_{4}=\frac{1}{r}\sum_{w=1}^{r}Yy_{w}$$ (26)

Here, *r* as the entire count of images and *f*_4_ implies mean.

*(ii) Variance*

It refers the square of standard deviation with the values of input and output image that is computed by,

$$f_{5}=Va^{2}=\frac{1}{r}\sum_{w=1}^{r}\left(Yy_{w}-f_{4}\right)$$ (27)

Here, *f*_5_ specifies variance.

*(iii) Standard deviation*

It indicates the square root of variance that is evaluated by,

$$f_{6}=\sqrt{\frac{1}{r}\sum_{w=1}^{r}\left(Yy_{w}-f_{4}\right)}$$ (28)

Here, the standard deviation is represented by *f*_6_.

*(iv) Kurtosis*

It is referred to describe the image distribution approximately with the mean, which is calculated by,

$$f_{7}=\frac{\sum {\left(f_{4}-\bar{f_{4}}\right)}^{4}}{rf_{7}}$$ (29)

Here, *f*_7_ as kurtosis.

*(v) Skewness*

It measures the distorted image from the normal image and it is expressed by,

$$f_{8}=\frac{\sum_{w}^{r}\left(l_{r}-\bar{f_{4}}\right)}{(r-1)*f_{6}}$$ (30)

Here, ℓ_*r*_ is signified as a random image.

Thus, the extracted features are illustrated by,

$$F_{x}=\{f_{3},f_{4},f_{5},f_{6},f_{7},f_{8}\}$$ (31)

#### 4.2.5 COVID-19 detection

The extracted feature vectors *F*_*x*_ are forwarded to the COVID-19 detection, which is done employing DNFN that is trained by utilizing RNBJSO. Here, RNBJSO is obtained by the incorporation of RNBO and JSO, where RNBO is the formation of ROA and NBO. The architecture of DNFN is delivered in the below sub-fragment.

**a) Structure of DNFN**

The structure of DNFN [25] comprises input layer, hidden layer and output layer, where the hidden layer if for learning and validation purposes. The input layer is based on numerous factors and the fuzzification values in this module. The hidden layers like rule, normalization and de-fuzzification layer, which is considered as the output layer.

Every input or output parameter is merged to a certain detail processing unit for every layer. The degree of every input is in the range of 0 and 1. The arithmetic expression is computed by,

$$PP_{1,\infty }=\delta_{\mu }S_{\infty }(uu)\;\mathrm{Or}\;PP_{1,\infty }=\delta_{\mu }T_{\infty -2}(\nu \nu ),\;\forall \infty =1,2,3,4.$$ (32)

Here, *uu*, νν are illustrated as consequent, *S*_∞_ and *T*_∞−2_ represents the antecedent functions and *PP*_1,∞_ indicates the degree of membership in layer 1.

The layer 2 that is the rule base layer employed to describe the set of rules. Here, each entity increased the linguistic variable to assure the membership degree, which is formulated as,

$$PP_{2,\infty }=\varpi \varpi_{\infty }=\delta_{\mu }S_{\infty }(uu)\delta_{\mu }T_{\infty -2}(\nu \nu ),\forall \infty =1,2.$$ (33)

Here, ϖϖ_∞_ implies weight of generic network factor. Layer 3 contracts with normalization wherein every entity examines the ratio of strength with the summation of firing strength that is computed by,

$$PP_{3,\infty }=\varpi \varpi_{\infty }=\frac{\varpi \varpi_{\infty }}{\varpi \varpi_{1}+\varpi \varpi_{2}},\forall \infty =1,2.$$ (34)

The de-fuzzification that is layer 4, wherein every rule consequents are examined to indicate an entire effect on the resultant, which is formulated by,

$$PP_{4,\infty }=\varpi \varpi_{\infty }y_{ii}=\varpi \varpi_{\infty }(m_{\infty }uu+n_{\infty }\nu \nu +o_{\infty }),\forall \infty =1,2.$$ (35)

where, *m*,*n*,*o* are signified as the consequent set of factors. Moreover, the last layer is called as summation layer determines the progression of the last result computation that is formulated by,

$$PP_{5,\infty }=\sum_{\infty }\bar{\varpi \varpi_{\infty }}y_{ii}=\frac{\sum_{\infty }\varpi \varpi_{\infty }y_{ii}}{\sum_{\infty }\varpi \varpi_{\infty }}$$ (36)

**Fig 5:**
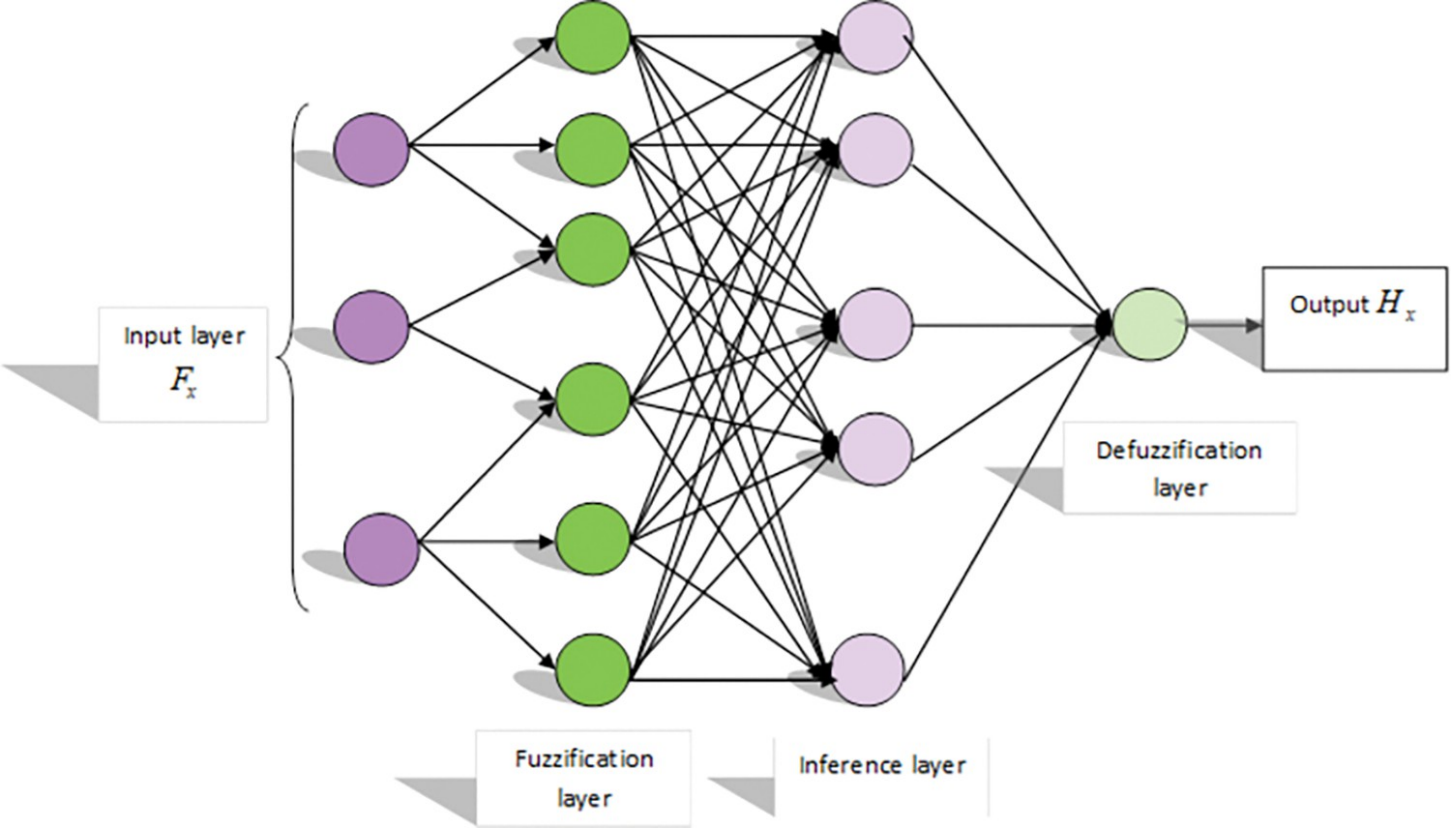
Architectural view of DNFN.

The factors employed in this structure are initially examined with random numbers and tuned with the training algorithm for supreme resultants of the model. The detected output is symbolized as *H*_*x*_. The architecture of DNFN is illustrated in Fig 5.

**b) Training Algorithm of RNBJSO**

ROA [27] is enthused of parasitic traits of remora as well as it furnishes an extremely promising position and supreme competitive ability. NBO [26] is inspired by the traits of Namib beetles, which have a clear technique to generate water and hence deduce data space. JSO [14] is based on the inspiration of the behaviour and movement of jellyfish in the ocean. Here, RNBJSO is obtained by the incorporation of RNBO and JSO, where RNBO is the formation of ROA and NBO. Here, DNFN is trained by the model RNBJSO to obtain the utmost results.

### Solution encoding

In a given search space (Δ), the solution encoding is employed to obtain the utmost solution, which is illustrated by,

Δ=[1×ℵ] (37)

where, the learning factor of DNFN is ℵ.

### Fitness Measure

It is utilized to find the optimum solution using the expression of the difference between the output target and the outcome of DNFN that is computed by,

$$A=\frac{1}{b}\sum_{a=1}^{b}{\left[\phi_{a}-H_{a}\right]}^{2}$$ (38)

where, the target output is ϕ_*a*_, the outcome of DNFN is *H*_*a*_ and the fitness is A.

### Algorithmic Steps

The algorithmic stages of RNBJSO are deliberated in the beneath fragment.

**Step 1: Initialization**

The first phase is to initialize the problem of the population in every solution computed by,

$$B=\{B_{1},B_{2},\ldots ,B_{b}\ldots ,B_{c}\}$$ (39)

Here, *B*_*b*_ signifies *b*^*th*^ candidate solution, *c* implies the count of variables and population is indicated by *B*.

**Step 2: Examine the fitness**

It is employed to examine an optimal solution and to obtain the utmost solution by employing Eq (38).

**Step 3: Appropriate position to collect the water**

In order to solve every solution in random space, the beetle is to be initialized utilizing the intent function, which has the extreme ability to collect the water and to moisture by the assessment of high values from every beetle. From this perspective, the beetle is placed in the finest area that may be striking for other beetles direct them to collect the water in the regions. In every region, the beetle *B*_*b*_ is positioned with the capability for propelling several beetles which is expressed by,

$$E_{b}=E_{e}.\sin(\frac{\mu (B_{b})-\hbar_{n}}{\mu_{g}-\mu_{e}}.\frac{\pi }{2})$$ (40)

Here, E_*b*_ is denoted as the ability of beetles count in a region, E_*e*_ is signified as high capability of beetles. μ(*B*_*b*_) is implied as the competence of beetle and μ_*e*_ and μ_*g*_ are the minimal and maximal abilities of beetles. Here, E indicates the entire population of beetles that is seeking water. A non-linear value is increased by the benefits from zero and E_*e*_.


$$E=\sum_{b=1}^{c}E_{b}=E_{1}+E_{2}+\ldots +E_{c}$$ (41)

**Step 4: Examine the migration to wet areas**

Every beetle needs to elect the area with enough wetness for finding the water, which is assumed as every beetle has attraction similar to wetness surrounded by the area. Hence, beetle attracts towards this area, where this type receives the wetness deduces with the escalation in distance. Assume beetle in single area *B*_*b*_ and in problem search space *B*_*a*_. The count of beetles tends to migrate towards the beetle *B*_*b*_. The distance amid two beetles is computed as,

$$\varphi_{ba}=\|B_{b}-B_{a}\|=\sqrt{\sum_{b=1}^{c}{\left(B_{b,c}-N_{a,c}\right)}^{2}}$$ (42)

$$D(\Phi )=\propto *D_{0}.\exp(-\varphi_{ba}^{\partial })$$ (43)

Here, *D*_0_ is symbolized as the amount of initial humidity equivalent to 2 and *D*(Φ) is indicated by the amount of wetness, where *B*_*a*_ is from regions of beetle *B*_*b*_, ∂ is implied as power, and φ_*ba*_ is signified as the distance. The co-efficient of rising humidity in terms to the proximity is examined with increased iteration and it will be altered by the trait from local to global as illustrated by,

$$\propto =o_{e}-o_{0}.(1-\frac{ℜ}{{ℜ}_{e}}).rand(0,1)$$ (44)

Here, the present iteration count is ℜ and ℜ_*e*_ is signified as maximal iteration count, *o*_0_ is an initial coefficient of humidity and ℑ is illustrated as humidity coefficient by beetles adjacent to regions with maximum humidity.

An attraction system of one to another beetle in current position and coefficient of wetness are employed and it is formulated by,

$$B_{a}^{new}=B_{a}^{Ζ}+D.(B_{b}-B_{a}^{Ζ})+levy$$ (45)

From remora, the expression is employed for further process that is elucidated as,

$$P^{B+1}=P^{B}+T$$ (46)

here,

$$T=\Omega *(P^{B}-M*P_{best})$$ (47)

Ω=2*L*rand(0,1)−L (48)

Assume,

$$P^{B+1}=B_{a}^{new}$$ (49)

$$P^{B}=B_{a}^{Ζ}$$ (50)

$$P_{best}=B_{a}^{best}$$ (51)

Then, Eq (46) becomes

$$B_{a}^{new}=B_{a}^{Ζ}+(2*L*rand(0,1)-L)*(B_{a}^{Ζ}-M*B_{a}^{best})$$ (52)

The upgrade expression of RHBO is computed as,

$$B_{a}^{new}=\frac{\left(2rand(0,1)-1)L*M*B_{a}^{best}*(1-D)+[D*B_{b}+levy](1+2*L*rand(0,1)-L\right)}{2*L*rand(0,1)-L+D}$$ (53)

Subtracting $B_{a}^{old}$ on both sides, then the equation becomes,

$$B_{a}^{new}-B_{a}^{old}=\frac{\left(2rand(0,1)-1)L*M*B_{a}^{best}*(1-D)+[D*B_{b}+levy](1+2*L*rand(0,1)-L\right)}{2*L*rand(0,1)-L+D}-B_{a}^{old}$$ (54)

By integrating the upgrade equation of JSO, the further process will be attained the updated solution for this developed model, which is calculated by,

$$A_{p}(q+1)=A_{p}(q)+rand(0,1)*A^{*}-\alpha \;rand(0,1)*\lambda$$ (55)

Assuming,

$$A_{p}(q+1)=B_{a}^{new}$$ (56)

$$A_{p}(q)=B_{a}^{old}$$ (57)

$$A^{*}=B_{a}^{best}$$ (58)

Substituting Eqs (56), (57) and (58) in Eq (55),

$$B_{a}^{new}=B_{a}^{old}+rand(0,1)*B_{a}^{best}-\alpha \;rand(0,1)*\lambda$$ (59)

$$B_{a}^{old}=B_{a}^{new}-rand(0,1)*B_{a}^{best}+\alpha \;rand(0,1)*\lambda$$ (60)

Substitute Eq (60) in Eq (54),

$$\begin{array}{l} B_{a}^{new}=\frac{\left(2rand(0,1)-1)L*M*B_{a}^{best}*(1-D)+[D*B_{b}+levy](1+2*L*rand(0,1)-L\right)}{2*L*rand(0,1)-L+D}\\ \;-[B_{a}^{new}-rand(0,1)*B_{a}^{best}+\alpha rand(0,1)*\lambda ] \end{array}$$ (61)

$$\begin{array}{l} B_{a}^{new}+B_{a}^{new}=\frac{\left(2rand(0,1)-1)L*M*B_{a}^{best}*(1-D)+[D*B_{b}+levy](1+2*L*rand(0,1)-L\right)}{2*L*rand(0,1)-L+D}\\ \;+rand(0,1)*B_{a}^{best}-\alpha \;rand(0,1)*\lambda \end{array}$$ (62)

$$B_{a}^{new}=\frac{\begin{array}{l} \left(2rand(0,1)-1)L*M*B_{a}^{best}*(1-D)+[D*B_{b}+levy](1+2*L*rand(0,1)-L\right)\\ \;+[rand(0,1)*B_{a}^{best}-\alpha \;rand(0,1)*\lambda ][2*L*rand(0,1)-L+D] \end{array}}{4*L*rand(0,1)-L+D}$$ (63)

Here, the remora parameters are signified as λ,*L*,*M*, where $L=2*(\frac{q}{Max_{iter}})$, the best position is indicated as $B_{r}^{best}$, $B_{a}^{new}$ and $B_{a}^{Ζ}$ are new and current positions of beetle and random number is signified as *rand*(0,1). The random vector *levy* is computed by,

$$levy=\frac{w}{{\left|L\right|}^{\frac{1}{\alpha }}}|\frac{\Gamma (1+\alpha )\cdot \sin(\frac{\pi }{2})\alpha }{\Gamma (\frac{1+\alpha }{2})\cdot \alpha_{2}^{\frac{\alpha -1}{2}}}|$$ (64)

where, β is indicated as constant equivalent to 1.5, *w* and *L* are random vectors in the range of (0,1).

**Step 5: Examine population mass and movement towards wet mass**

By sensing the fragrance of high wetness, the beetles are capable to predict the areas. In order to enhance this behaviour, the center of gravity and the wet locations must be utilized. The search space of beetles is around the gravity point and finest solution. The quantity of water and wet is considered for every beetle that can be employed from this center of gravity for extreme search. Hence, the regions with a high chance to predict the water is illustrated as,

$$B_{a}^{new}=B_{a}^{Ζ}+rand.(B^{*}-\bar{B})+levy$$ (65)

**Step 6: Examine the population’s hunting and removal**

Beetles are ready to return to their own place after watering progress is performed on hill and wetness also collected from air. Here, few of them are hunted by lizards and this is probable to furnish an opportunity to remove a beetle with a negligible of solution. Normally, impossible solutions are neglected from the population and it is extremely prone to dispose and hunt. Hence unsystematic solutions are created in the space of problems.

**Step 7: Termination**

This progress will be till it attains proper solution with utmost outcomes and the pseudo-code of RNBJSO is described in algorithm 2.

**Algorithm 2. Pseudo code of RNBJSO**.

SL. No Pseudo code of RNBJSO

1 **Input**: Population *B*, Maximum iteration *Max*_*iter*_

2 **Output**: $B_{a}^{new}$

3 **Begin**

4 Initializing the population

5 Compute fitness value by Eq (26)

6 *While*(ℜ< = ℜ_*e*_) *do*

7 $\mu_{e}=e(\mu ),\mu_{g}=g(\mu )$

8 Determine the initial coefficient of humidity by Eq (44)

9 *for b* = 1 to *c do*

10  Evaluate the ability for getting several beetles by Eq (38)

11 *end for*

12 *forb* = 1 to *c do*

13 *for a* = E_*b*_ to *c do*

14 Searching around *B*_*b*_

15  *end for*

16 *end for*

17 *for b* = 1 to *c do*

18  *for a* = 1 to *c do*

19 Evaluate web areas and upgrade solution by Eqs (42), (43) and (63)

20  *end for*

21 *end for*

22 *for b* = 1 to *c do*

23  Evaluate population mass and move towards wet mass by Eq (65)

24  *end for*

25 ℜ = ℜ+1

26 *end while*

27 Return

28 Terminate

### 4.3 Cloud layer

**Fig 6:**
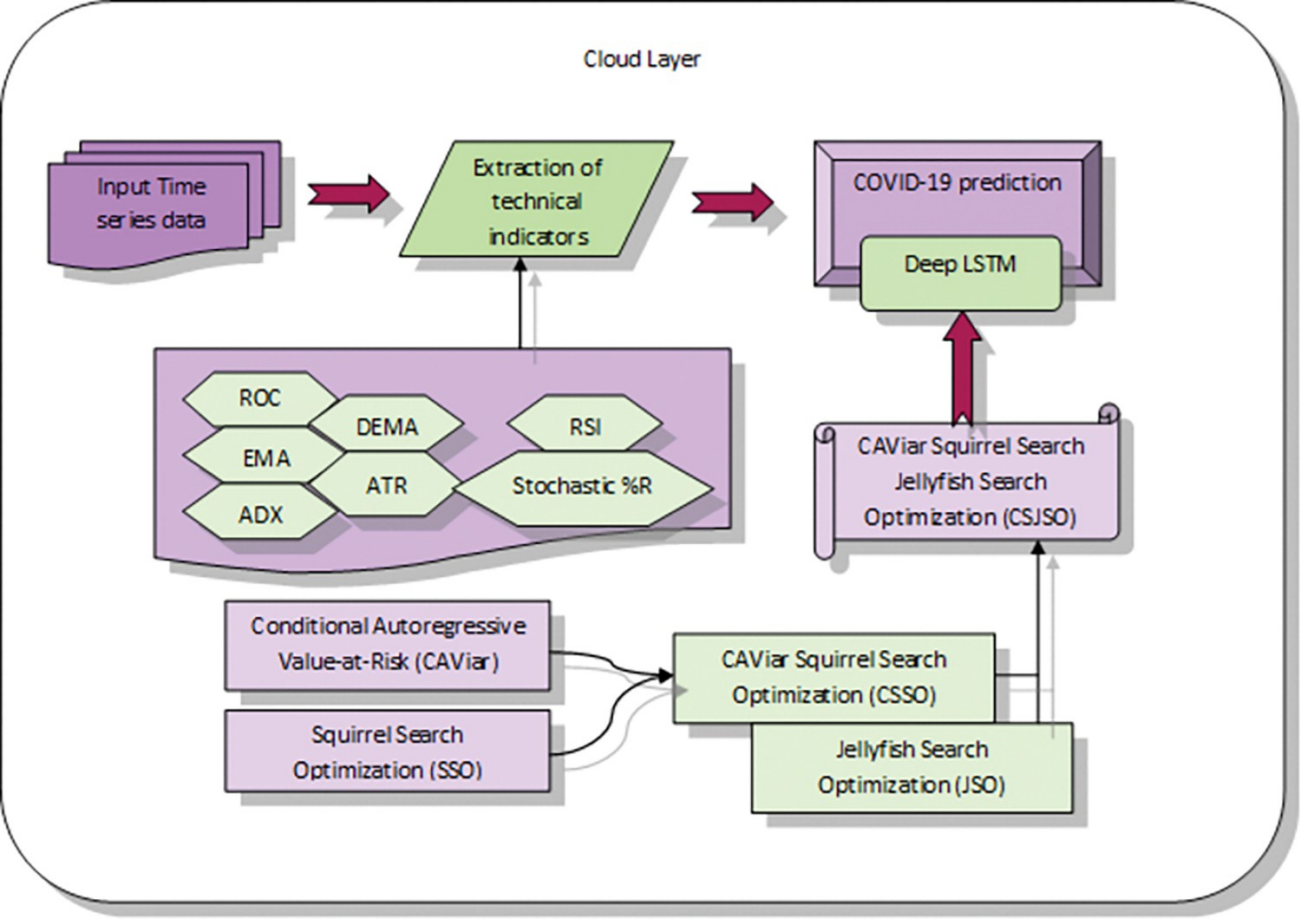
Modelled view of cloud layer.

Finally, in the cloud data center, the COVID-19 prediction at a particular region is performed in the cloud layer. After that, the COVID prediction is conducted by employing the following phases. At first, the input time series data is allowed to the technical indicators extraction unit. Here, the technical indicators employed such as the Exponential Moving Average (EMA), Relative Strength Index (RSI), Average Directional Movement Index (ADX), and Average True Range (ATR), stochastic %R, Double Exponential Moving Average (DEMA), and Rate of Change (ROC) [17,28]. Then, the extracted indicators are carried out to the data augmentation unit. Afterwards, the augmented data is forwarded to the COVID-19 prediction, wherein the prediction is accomplished by Deep LSTM [29] trained employing CSJSO. Fig 6 illustrates the model of the cloud layer.

#### 4.3.1 Data acquisition

Assume the standard database *C* with *n* number of time series data that is determined by,

$$C=\{C_{1},C_{2},\ldots C_{m},\ldots C_{n}\}$$ (66)

Where, *C*_*m*_ is considered as the input for the further process at cloud layer.

#### 4.3.2 Technical indicators extraction

The input time series data *C*_*m*_ is forwarded to the extraction unit in order to attain the appropriate technical indicators. Here, the technical indicators extracted such as EMA, RSI, ADX, ATR, stochastic %R, ROC and DEMA [17,28], which are briefly explained in the sub-fragments.

**a) EMA**

It is a moving average, where it considers the weight to determine the resultants of future prediction. EMA is formulated by,

$$i_{1}=\sum_{y=0}^{z-1}O_{y}Q_{x-y}$$ (67)

where, *O* is indicated as weight, and EMA is signified as *i*_1_.

**b) RSI**

It refers the fault of the predicted case in regards to the last closing cases of COVID-19, which is computed by,

$$i_{2}=100-\frac{100}{\left(1+f_{1}(j^{+})/f_{1}(j^{-})\right)}$$ (68)

where, *j*^+^ as the increased cases, and *j*^−^ as decreased cases. RSI is signified by *i*_2_.

**c) ADX**

It is utilized to examine the entire strength of prediction and needs a series of evaluation due to numerous lines in the indicator that is evaluated by,

$$i_{3}=\frac{(M\times 13)+N}{14}$$ (69)

Here, the preceding ADX is signified as M, present ADX is signified as N and ADX is implied as *i*_3_.

**d) ATR**

It provides information of the degree of transmission of the cases of COVID-19. ATR is calculated by,

$$i_{4}=i_{1}(\max(T_{x}-U_{x}|T_{x}-Q_{x-1}),|U_{x}-Q_{x-1}|))$$ (70)

where, higher and lower number of COVID-19 cases is symbolized as *T*_*x*_,*U*_*x*_, edifies the individuals affected per day is specified as *V*_*p*_, || is symbolized as appropriate value, and *i*_4_ is implied as ATR.

**e) Stochastic %D**

It furnishes the turn round data of time series that represents the individuals who are infected with COVID-19 or not. It is determined by 3 days EMS of stochastic %K with a period of days.

$$i_{5}=i_{1}^{3}(\%V_{x})$$ (71)

where, *i*_5_ as stochastic %D.

**f) DEMA**

It is employed to reduce the entire lag that occurred in the normal moving averages and it is the mean of COVID-19 cases to provide weight for the new case. DEMA is formulated by,

$$i_{6}=(2*B(z))-(B(B(z)))$$ (72)

where, *i*_6_ is symbolized as DEMA, B is signified as EMA, and *z* is referred as time.

**g) ROC**

It is employed to measure the rate of regular change with prior time interval of prediction that is illustrated by,

$$i_{7}=\frac{X(t)}{X(t-g)}*100$$ (73)

where, X is indicated as the prediction of COVID-19, X(*t*) is signified as the prediction of COVID-19 at *t* and X(*t*−*g*) is illustrated as the change of prediction at the time *t*. The ROC technical indicator is specified as *i*_7_.

Finally, the extracted technical indicators *I*_*m*_ are represented as,

$$I_{m}=\{i_{1},i_{2},.....i_{7}\}$$ (74)

#### 4.3.3 Data augmentation

The extracted technical indicators *I*_*m*_ are allowed to the data augmentation to perform the oversampling by dividing the data by means of class labels to generate the remaining samples, which are created to improve the dimensions of the data. The outcome of data augmentation is signified as *J*_*m*_.

#### 4.3.4 COVID-19 prediction using Deep LSTM

The augmented data *J*_*m*_ is allowed to the COVID-19 prediction in order to predict the affected patient utilizing Deep LSTM. Here Deep LSTM is trained by CSJSO. The structure of Deep LSTM and the training algorithm of CSJSO are described in the below fragments.

**a) Deep LSTM**

Deep LSTM [29] architecture has LSTM layers, dropout layers and dense layer. The primary network of sequential learning is the Recurrent Neural Network (RNN). In few layers, the recurrent neurons result the response as *X*_τ_ are analyzed in terms of input E_τ_ and the response E_τ−1_ from preceding slots. It is expressed by,

$$X_{\tau }=\varepsilon (\psi_{\omega X}E_{\tau }+\psi_{XX}E_{\tau -1}+\xi_{X})$$ (75)

LSTM is the improvised description of RNN, which learns about long-range dependencies. It has input, forget, cell and output gate and output response. The flow of information is directed by the input and forget gate. The information form the cell is passed to output managed by the output gate. The memory cell contains a self-connected recurrent edge of weight, which ensures gradient and may allow various time stages with lack of exploration. Therefore, it overcomes the issues of RNN by vanishing the gradient effect. For entire LSTM neurons in a few layers at time *τ*, the recursive activation computation of units is determined as,

$$W_{\tau }=\phi (\psi_{\omega W}E_{\tau }+\psi_{XW}X_{\tau -1}+\psi_{OW}O_{\tau -1}+\xi_{W})$$ (76)

$$Y_{\tau }=\phi (\psi_{\omega Y}E_{\tau }+\psi_{XY}X_{\tau -1}+\psi_{OY}O_{\tau -1}+\xi_{Y})$$ (77)

$$O_{\tau }=Y_{\tau }⊕O_{\tau -1}+W_{\tau }⊕\tanh(\psi_{\omega O}E_{\tau }+\psi_{XO}X_{\tau -1}+\xi_{Ι})$$ (78)

$$Z_{\tau }=\phi (\psi_{\omega Z}E_{\tau }+\psi_{XZ}X_{\tau -1}+\psi_{OZ}O_{\tau -1}+\xi_{Z})$$ (79)

$$X_{\tau }=Z_{\tau }⊕\tanh(O_{\tau })$$ (80)

**Fig 7:**
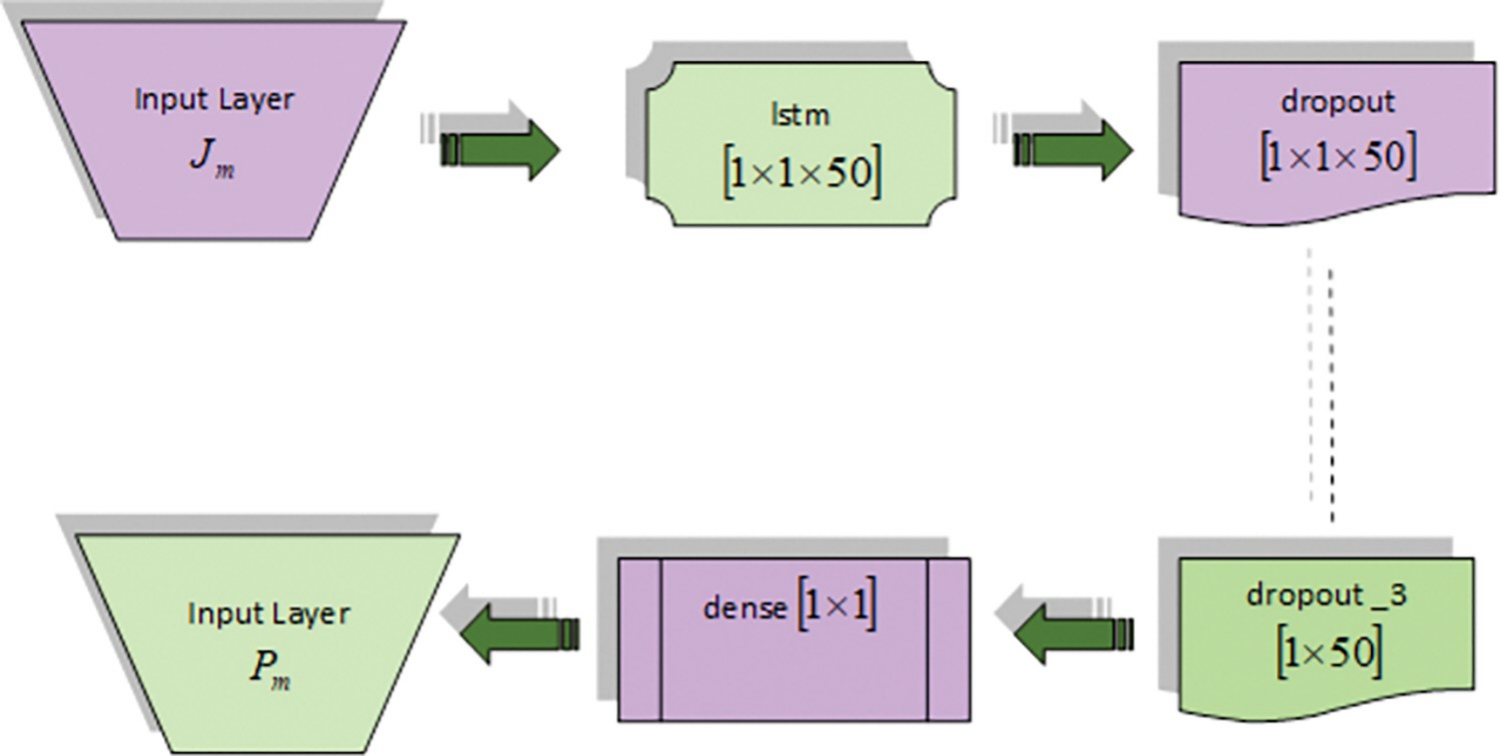
Architecture of Deep LSTM.

Here, the element-wise product is ⊕,bias is ξ_*Z*_. To compute the outcome, bidirectional LSTM is utilized to provide the details of the future and past. The predicted data is represented by *P*_*m*_. The architecture of Deep LSTM is in Fig 7.

**b) Training Algorithm of proposed CSJSO**

Deep LSTM is trained by the devised technique CSJSO. Here, CSJSO is achieved by the concatenation of CSSA and JSO, where CSSA is blended and formed by the amalgamation of CAViar and SSA. The training algorithm of the presented model CSJSO is already explained in segment 4.1.1.

Position Encoding

In a given search space (ς), position encoding is utilized to examine the optimal position for extreme solution utilizing CSJSO, which is formulated by,

ς=[1×κ] (81)

Here, the learning factor of CSJSO is κ

Fitness Measure

It is utilized to attain the supreme outcome of CSJSO and it depicts the extraction since CSJSO is employed in this training stage that is illustrated as,

$$Y=\frac{1}{n}\sum_{m=0}^{n}{\left[\zeta_{m}-P_{m}\right]}^{2}$$ (82)

Here, Y defines the fitness measure, the output target symbolizes ζ_*m*_ and *P*_*m*_ implies the resultant of Deep LSTM.

## 5. Results and discussion

The results of the presented model CSJSO_Deep LSTM is deliberated and discussed with the implementation, description of database, metrics and the analysis of comparative schemes of routing and CSJSO_Deep LSTM.

### 5.1 Experimental setup

The CSJSO_Deep LSTM is experimentally conducted with the PYTHON tool in Windows 10 OS.

### 5.2 Experimental outcomes

**Fig 8:**
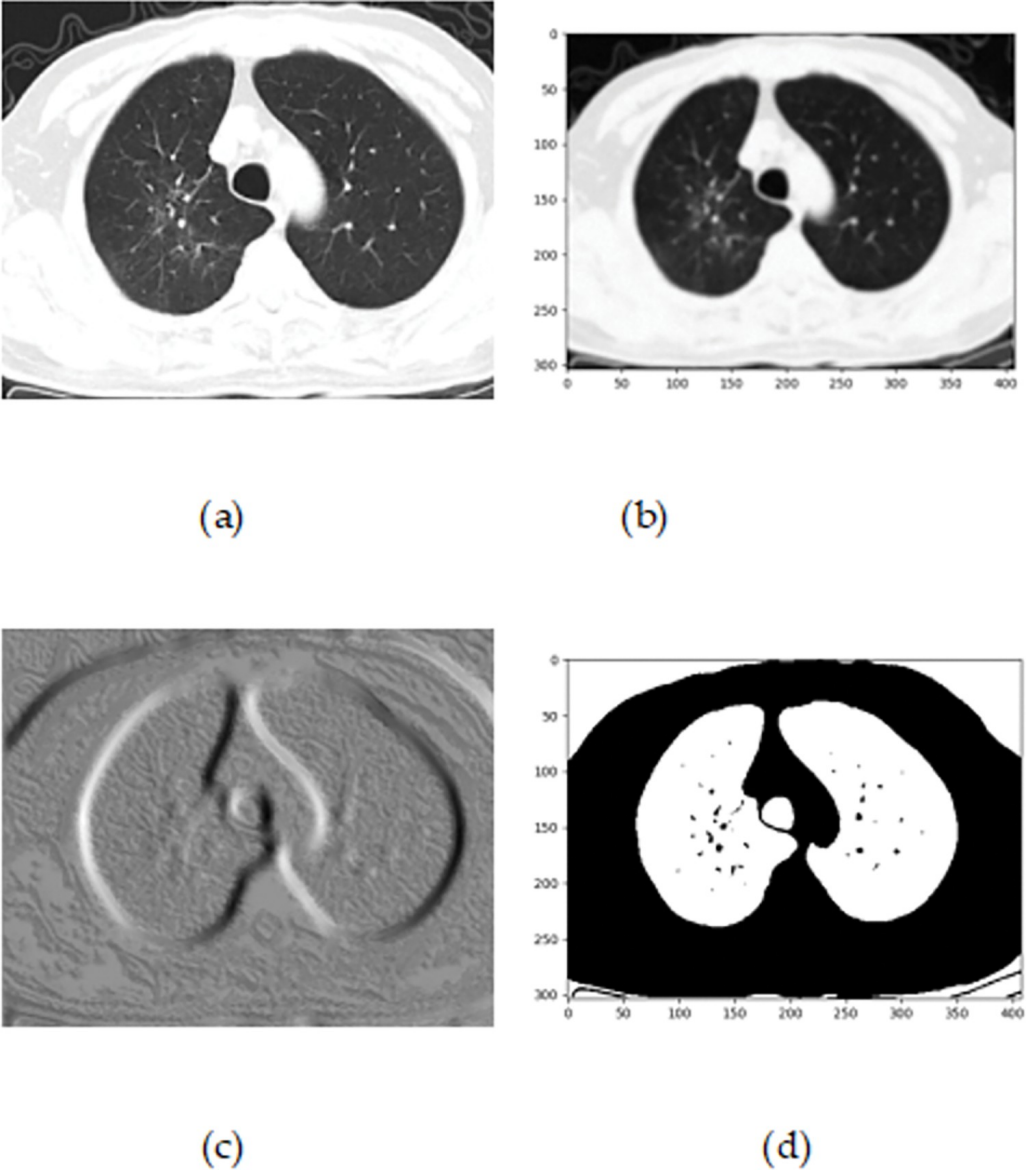
Experimental outcomes of CSJSO_Deep LSTM a) Input image, b) Pre-processed image c) Extracted image and d) Segmented image.

The resultants of CSJSO_Deep LSTM are designed in Fig 8. In Fig 8A), the input image is indicated and in Fig 8B), pre-processed image is represented. Fig 8C) enumerates extracted image and Fig 8D) elucidates segmented image.

### 5.3 Simulation parameters

**Fig 9:**
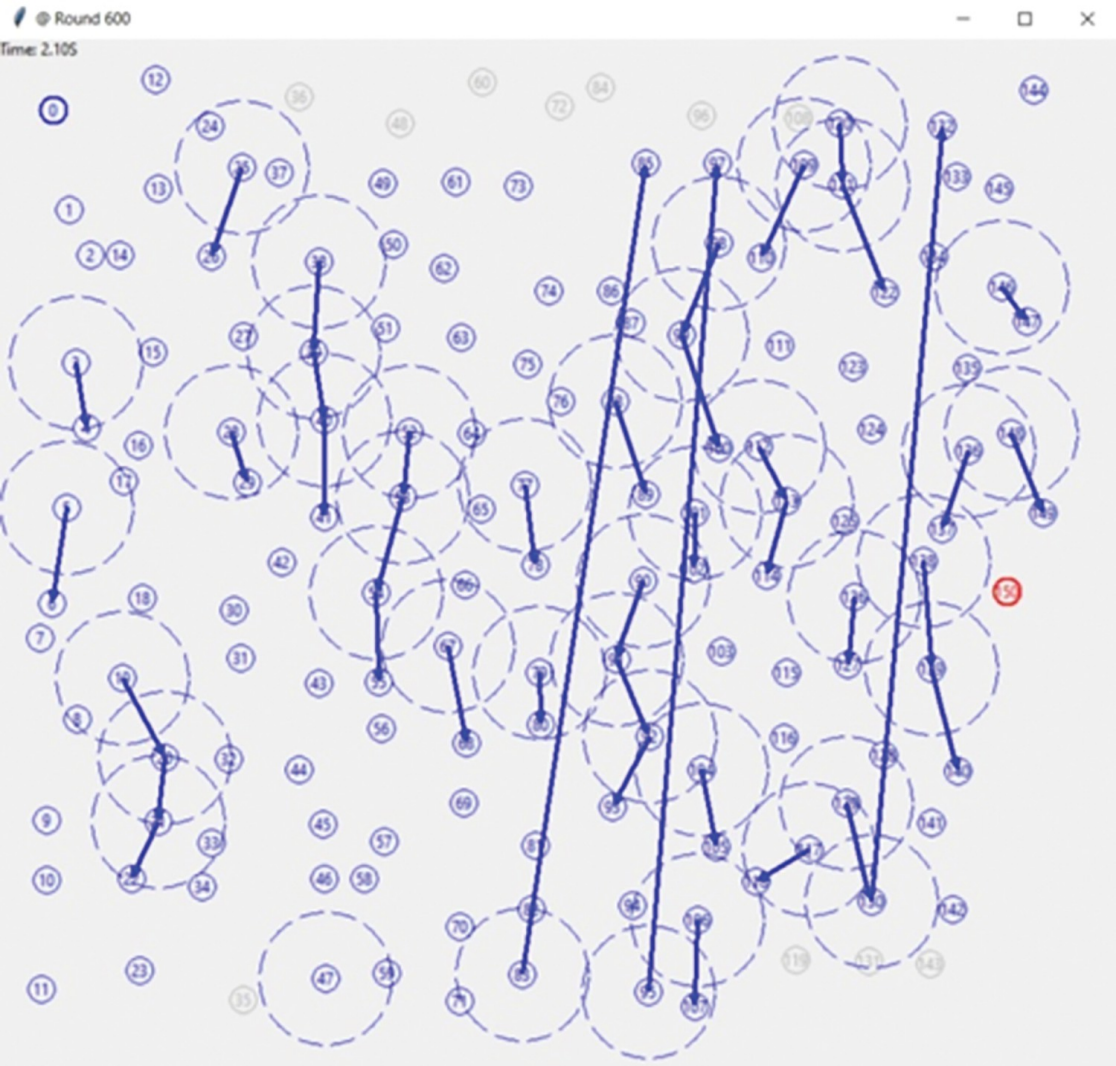
Simulation parameter.

The simulation parameter of CSJSO_Deep LSTM is designed and in Fig 9.

The description of simulation parameters is described in Table 1.

**Table 1:** Simulation parameters.

X	100
Y	100
Initial Energy	1

### 5.4 Dataset description

The description of database-1 and database-2 employed for CSJSO_Deep LSTM is described in this section.

#### 5.4.1 Database-1: Novel coronavirus

This database [18] comprises of COVID-19’s confirmed cases, death cases and recovered cases. The file format of this database is in CSV format. This source of data is created by Johns Hopkins University Center for Systems Science and Engineering (JHU CCSE) from numerous resources. The fields available in every data are Country/Region, Province/State, Last Update, Suspected, Confirmed, Deaths, and Recovered.

#### 5.4.2 Database-2: COVID-CT

The COVID-CT [30] comprise 349 images with medical diagnose of COVID-19 from 216 patients and 463 non-COVID patients. The database is in zip format. The images gathered from COVID-19 relevant from medRxiv, NEJM, JAMA and so on. The data contains patient information, patient ID, image caption, and DOI.

### 5.5 Performance measures

In this fragment, the measures of CSJSO_Deep LSTM namely, MSE and RMSE for database-1 and accuracy, sensitivity, and specificity for database-2 are described.

#### 5.5.1 MSE

It referred as the mean squared variance amid the exact and the predicted value, which is formulated by Eq (79).

#### 5.5.2 RMSE

It is referred as the square root of MSE and it is computed as,

$$Y=\sqrt{\frac{1}{n}\sum_{m=0}^{n}{\left[\zeta_{m}-P_{m}\right]}^{2}}$$ (83)

#### 5.5.3 Accuracy

It is utilized for the evaluation of the possibility or exactness of the diagnose formulated by,

$$Acc=\frac{T_{P}+T_{N}}{T_{P}+T_{N}+Y_{P}+Y_{N}}$$ (84)

#### 5.5.4 Sensitivity

It is employed to examine the true positives calculated by,

$$Sen=\frac{T_{P}}{T_{P}+Y_{N}}$$ (85)

#### 5.5.5 Specificity

This computes the exact outcomes of true negative determined by,

$$Spec=\frac{T_{N}}{T_{N}+Y_{P}}$$ (86)

Here, true positive and true negative is indicated as T_P_, T_N_ and false positive and false negative is signified as Y_N_, Y_P_.

#### 5.5.6 Energy

Each node in IoT has prior energy, where the nodes are not rechargeable. When the data transmission is performed, the dissipation of energy at the transmitter is done by a power amplifier and radio electronics.

#### 5.5.7 Trust

In the trust model, Ξ_*kl*_ represents the trust amid *kt*^*h*^ and *l*^*th*^ nodes, which is computed by,

$$\Xi_{kl}=\frac{\Xi_{kl}^{\iota }+\Xi_{kl}^{Belief}}{2}$$ (87)

Here, trust among the beta distribution is signified as $\Xi_{kl}^{\iota }$.

### 5.6 Comparative methods

The assessment of the presented model CSJSO_Deep LSTM is examined with the preceding schemes like, Reinforcement learning [5], Auxiliary GAN [8], Deep learning [7] DNN [9], CSSA_Deep LSTM, and JSO_Deep LSTM in database-1 and Inf-Net [10], U-Net [1], Cascade CNN [11], Transfer learning [12], Auxiliary GAN [8], CSSA_Deep LSTM, and JSO_Deep LSTM in database-2. In the same way, the CSJSO_Deep LSTM is analyzed for routing with prior models, such as Fractional Artificial Bee Colony (FABC) [31], Multipath QoS Aware Routing Protocol (MMQARP) [32], Priority-based Congestion-avoidance Routing Protocol (PCRP routing) [33], Energy Efficient Routing Protocol using Dual Prediction Model (EERP-DPM) [34] and Autoregressive Squirrel Search (ArSS).

### 5.7 Comparative analysis

The evaluation is based on routing and the presented technique CSJSO_Deep LSTM is illustrated in the beneath sub-fragments.

#### 5.7.1 Assessment of CSJSO_Deep LSTM based on routing

**Fig 10:**
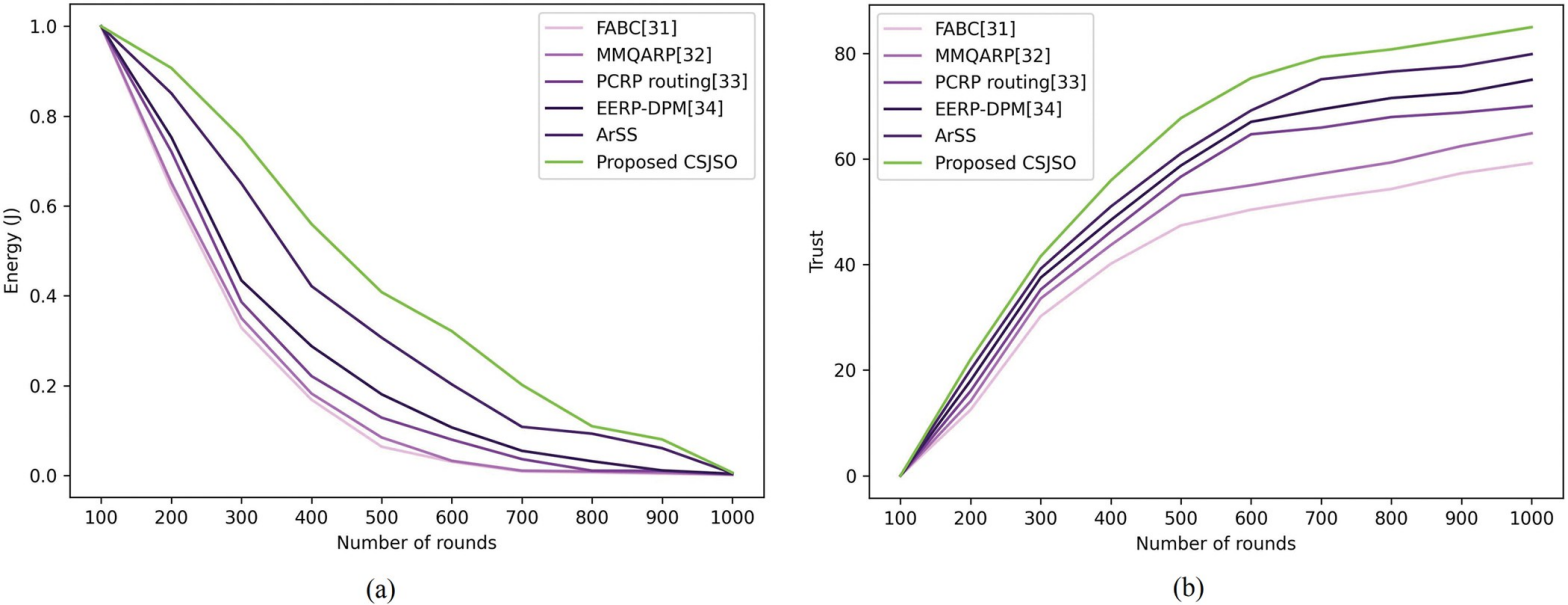
Evaluation of CSJSO_Deep LSTM based on routing a) Energy and b) Trust.

The evaluation of CSJSO_Deep LSTM based on routing altering rounds is designed in Fig 10. In Fig 10A), the CSJSO_Deep LSTM in terms of energy is illustrated. When the number of rounds is assumed as 1000, the CSJSO_Deep LSTM attained the energy as 0.006J, the existing models like FABC obtained 0.001, MMQARP achieved 0.001, PCRP routing acquired 0.003, EERP-DPM attained 0.004 and ArSS gained 0.005. In Fig 10B), the CSJSO_Deep LSTM by means of trust is devised. If the round is 1000, then the CSJSO_Deep LSTM gained 84.946, the traditional models namely, FABC as 59.195, MMQARP as 64.842, PCRP routing as 70.000, EERP-DPM as 79.836 and ArSS as 84.946.

#### 5.7.2 Analysis of CSJSO_Deep LSTM with database-1

The examination of CSJSO_Deep LSTM with database-1 comprises three setups namely, confirmed cases, death cases and recovered cases, which are clearly exploited below.

**a) Valuation of CSJSO_Deep LSTM with confirmed cases**

**Fig 11:**
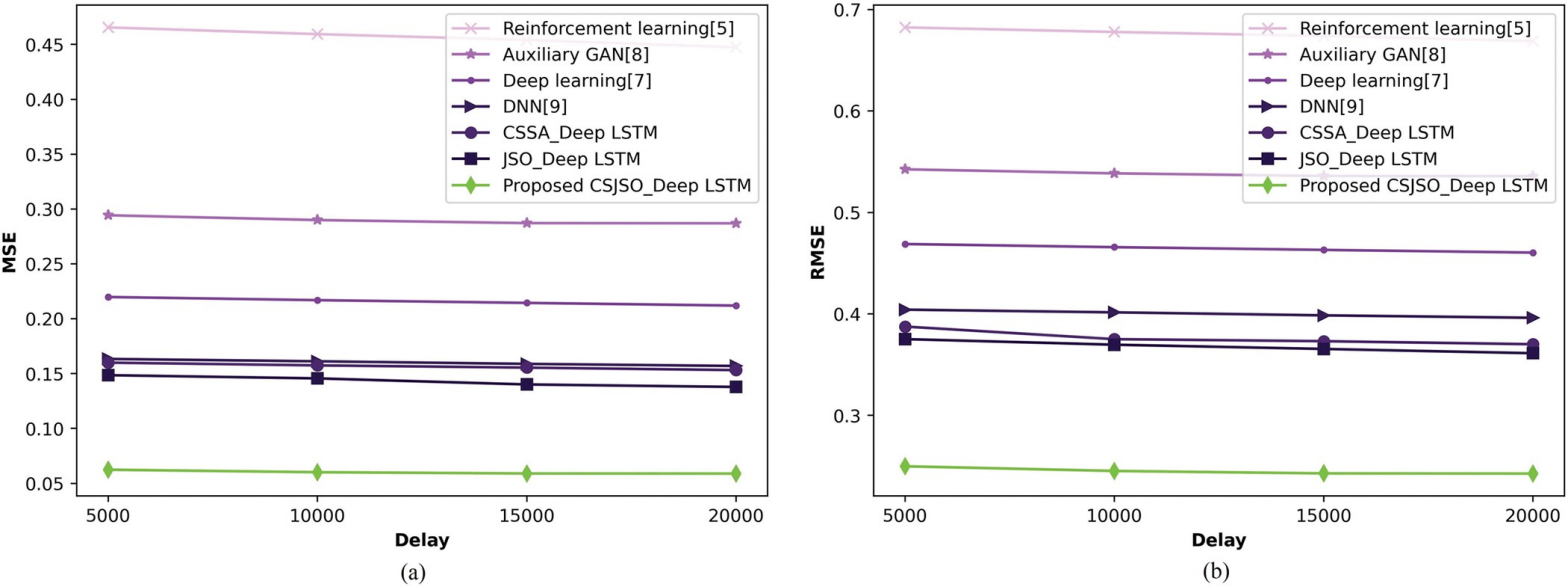
Valuation of CSJSO_Deep LSTM with confirmed cases a) MSE and b) RMSE.

Fig 11 enumerates the CSJSO_Deep LSTM on the basis of confirmed cases altering delay. In Fig 11A), the CSJSO_Deep LSTM in terms of MSE is represented. With the delay of 5000, the CSJSO_Deep LSTM obtained the MSE of 0.062, while the traditional models like Reinforcement learning, Auxillary GAN, Deep learning, DNN, CSSA_Deep LSTM, and JSO_Deep LSTM gained 0.466, 0.294, 0.220, 0.163, 0.160, and 0.149. Fig 11B) elucidates the RMSE with CSJSO_Deep LSTM. The CSJSO_Deep LSTM accomplished RMSE of 0.252, the conventional schemes namely, Reinforcement learning as 0.491, Auxiliary GAN as 0.425, Deep learning as 0.405, DNN as 0.354, CSSA_Deep LSTM as 0.388, and JSO_Deep LSTM as 0.375 with the delay of 5000.

**b) Valuation of CSJSO_Deep LSTM with death cases**

**Fig 12:**
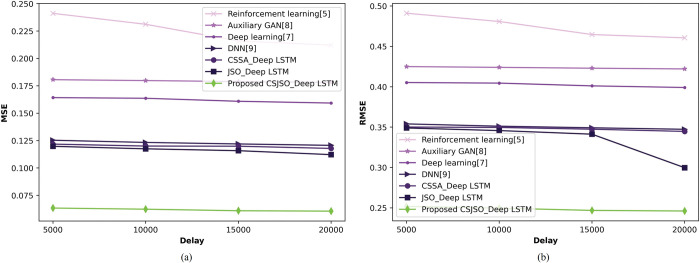
Valuation of CSJSO_Deep LSTM with death cases a) MSE and b) RMSE.

In Fig 12, the CSJSO_Deep LSTM in terms of death cases varying delay is illustrated. The CSJSO_Deep LSTM in regards of MSE is indicated in Fig 12A). When the delay is 5000, the CSJSO_Deep LSTM obtained the MSE of 0.250, the traditional models such as, Reinforcement learning as 0.682, Auxillary GAN as 0.542, Deep learning as 0.469, DNN as 0.404, CSSA_Deep LSTM as 0.122, and JSO_Deep LSTM as 0.120. In Fig 12B), the RMSE of CSJSO_Deep LSTM is specified. With the delay of 5000, the CSJSO_Deep LSTM gained RMSE of 0.061, the conventional schemes accomplished 0.273, 0.178, 0.175, 0.129, 0.350, and 0.349.

**c) Valuation of CSJSO_Deep LSTM with recovered cases**

**Fig 13:**
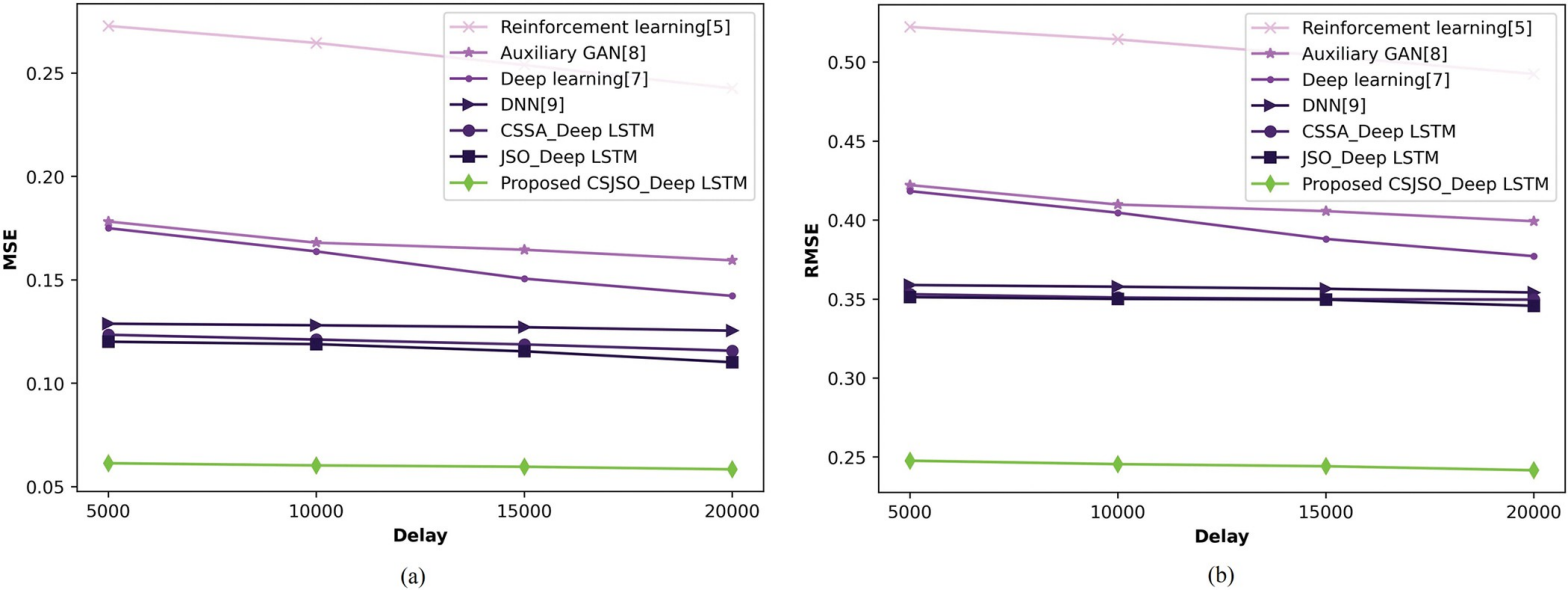
Valuation of CSJSO_Deep LSTM with recovered cases a) MSE and b) RMSE.

Fig 13 exploits the CSJSO_Deep LSTM in regards of recovered cases varying delay. In Fig 13A), the CSJSO_Deep LSTM with MSE is represented. If the delay is assumed as 5000, then the CSJSO_Deep LSTM acquired MSE of 0.063, while the prior techniques like, Reinforcement learning as 0.241, Auxillary GAN as 0.181, Deep learning as 0.164, DNN as 0.125, CSSA_Deep LSTM as 0.123, and JSO_Deep LSTM as 0.120. Fig 13B) designs the CSJSO_Deep LSTM with RMSE. The CSJSO_Deep LSTM of RMSE gained 0.248 and the preceding models gained 0.522, 0.422, 0.418, 0.359, 0.353, and 0.351.

#### 5.7.3 Analysis of CSJSO_Deep LSTM with database-2

The examination of CSJSO_Deep LSTM with database-2 is designed by altering the training set and k-set.

**a) Assessment of CSJSO_Deep LSTM altering training set**

**Fig 14:**
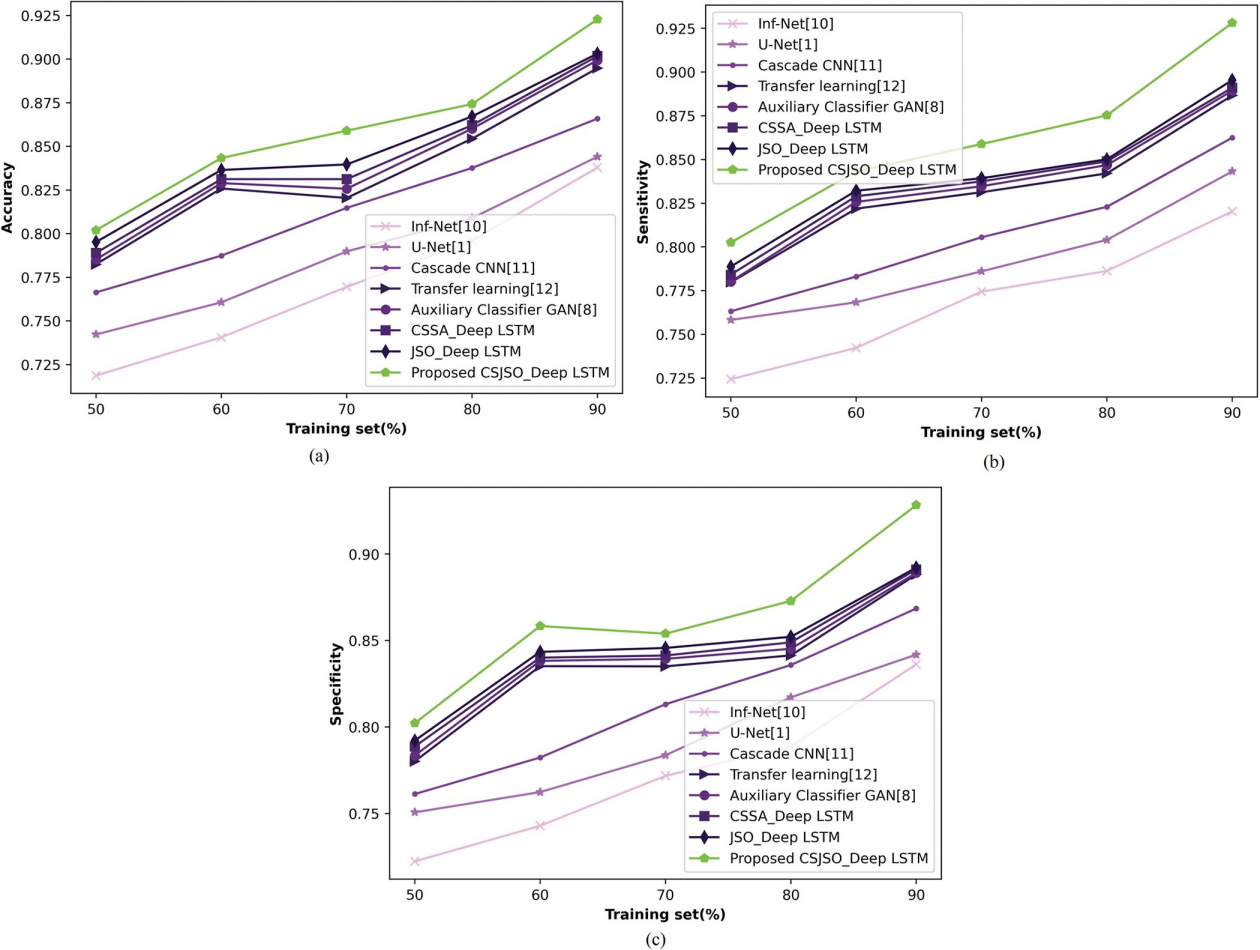
Examination of CSJSO_Deep LSTM altering training set on database-2 a) Accuracy, b) Sensitivity and c) Specificity.

In Fig 14, the CSJSO_Deep LSTM altering training set is designed. Fig 14A) represents the CSJSO_Deep LSTM of accuracy. With 90% of training set, the CSJSO_Deep LSTM gained accuracy of 0.923, while the performance gain of prior models namely, Inf-Net, U-Net, Cascade CNN, Transfer learning, Auxillary GAN, CSSA_Deep LSTM, and JSO_Deep LSTM obtained 9.199%, 8.529%, 6.166%, 3.031%, 2.600%, 2.275%, and 2.167%. In Fig 14B), the CSJSO_Deep LSTM of sensitivity is indicated. Assuming training set as 90%, the CSJSO_Deep LSTM gained 0.928, while the performance gain achieved 11.619%, 9.149%, 7.073%, 4.469%, 4.203%, 3.987%, and 3.556%. Fig 14C) signifies the specificity of CSJSO_Deep LSTM. By considering the training set = 90%, the CSJSO_Deep LSTM gained the specificity of 0.928, the performance gain of preceding techniques accomplished 9.915%, 9.312%, 6.440%, 4.324%, 4.203%, 3.987%, and 3.879%.

**b) Assessment of CSJSO_Deep LSTM varying K-set**

**Fig 15:**
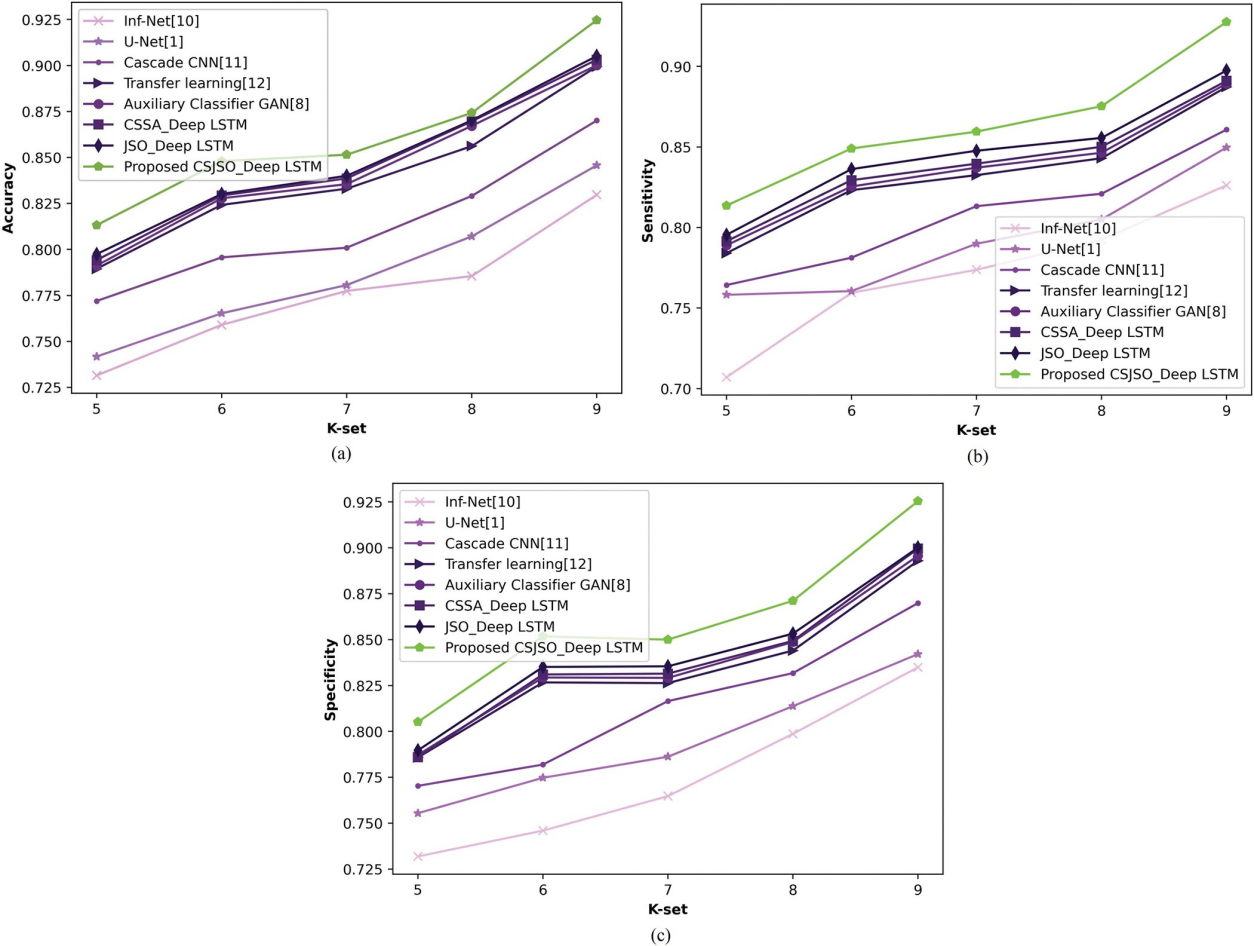
Examination of CSJSO_Deep LSTM altering K-set on database-2 a) Accuracy, b) Sensitivity and c) Specificity.

Fig 15 specifies the CSJSO_Deep LSTM altering K-set. In Fig 15A), the CSJSO_Deep LSTM in terms of accuracy is illustrated. If K-set = 9, the CSJSO_Deep LSTM observed an accuracy of 0.925, while comparing the performance gain of existing models obtained 10.274%, 8.536%, 5.900%, 2.727%, 2.703%, 2.378%, and 2.162%. Fig 15B) elucidates the CSJSO_Deep LSTM with sensitivity. With 9 as K-set, the CSJSO_Deep LSTM obtained the sensitivity of 0.928, the performance gain of traditional models accomplished 10.936%, 8.401%, 7.205%, 4.354%, 4.203%, 3.987%, and 3.341%. Fig 15C) indicates the specificity of CSJSO_Deep LSTM. Assuming K-set as 9, the CSJSO_Deep LSTM gained a specificity of 0.925, the performance gain while comparing achieved 9.784%, 9.004%, 6.003%, 3.512%, 3.135%, 2.703%, and 2.703%.

### 5.8 Comparative discussion

The comparative discussion of CSJSO_Deep LSTM is described in Tables 2–4. The metrics employed for routing is indicated in Table 2 namely, energy and trust obtained 0.006J, and 84.946. The evaluation measures utilized for CSJSO_Deep LSTM in database-1 are signified in Table 3 such as MSE and RMSE observed 0.062 and 0.252 in confirmed cases. The measures employed in database-2 are accuracy, sensitivity and specificity achieved 0.925, 0.928 and 0.925 as described in Table 4.

**Table 2:** Comparative discussion based on routing.

Number of Rounds	Metrics/ Methods	FABC	MMQARP	PCRP routing	EERP-DPM	ArSS	Proposed CSJSO_Deep LSTM
**Number of rounds = 1000**	*Energy (J)*	0.001	0.001	0.003	0.004	0.005	0.006
	*Trust*	59.195	64.842	70.000	74.980	79.836	84.946

**Table 3:** Comparative discussion on database-1.

Database-1	Metrics/ Methods	Reinforcement learning	Auxillary GAN	Deep learning	DNN	CSSA_Deep LSTM	JSO_Deep LSTM	Proposed CSJSO_Deep LSTM
**Confirmed cases**	*MSE*	0.466	0.294	0.220	0.163	0.153	0.138	**0.062**
	*RMSE*	0.491	0.425	0.405	0.354	0.370	0.361	**0.252**
**Death cases**	*MSE*	0.682	0.542	0.469	0.404	0.118	0.112	0.250
	*RMSE*	0.273	0.178	0.175	0.129	0.345	0.300	0.061
**Recovered cases**	*MSE*	0.241	0.181	0.164	0.125	0.116	0.110	0.063
	*RMSE*	0.522	0.422	0.418	0.359	0.350	0.346	0.248

**Table 4:** Comparative discussion on database-2.

Metrics/ Methods	Inf-Net	U-Net	Cascade CNN	Transfer learning	Auxillary GAN	CSSA_Deep LSTM	JSO_Deep LSTM	Proposed CSJSO_Deep LSTM
	**Training set = 90%**							
*Accuracy*	0.838	0.844	0.866	0.895	0.899	0.902	0.903	0.923
*Sensitivity*	0.820	0.843	0.862	0.887	0.889	0.891	0.895	0.928
*Specificity*	0.836	0.842	0.869	0.888	0.889	0.891	0.892	0.928
	**K-set = 9**							
*Accuracy*	0.830	0.846	0.870	0.899	0.900	0.903	0.905	**0.925**
*Sensitivity*	0.826	0.850	0.861	0.887	0.889	0.891	0.897	**0.928**
*Specificity*	0.835	0.842	0.870	0.893	0.896	0.900	0.900	**0.925**

## 6. Conclusion

In this research, a novel strategy is established for COVID-19 prediction-based fog-cloud named CSJSO. Here, CSJSO is the amalgamation of CSSA and JSO, where CSSA is blended by CAViar and SSA. This architecture comprises the healthcare IoT sensor layer, fog layer and cloud layer. In the healthcare IoT sensor layer the routing process is performed with the collection of patient’s health condition data and in the fog layer detection of COVID-19 is conducted employing Deep Neuro-Fuzzy Network (DNFN), which is trained by the proposed RNBJSO. Finally, the detection of COVID-19 is performed in the cloud layer employing Deep LSTM, which is trained utilizing CSJSO. The metrics employed for routing namely, energy and trust obtained 0.006J, and 84.946. The evaluation measures utilized for CSJSO_Deep LSTM in database-1, such as MSE and RMSE observed 0.062 and 0.252 in confirmed cases. The measures employed in database-2 are accuracy, sensitivity and specificity achieved 0.925, 0.928 and 0.925 in K-set. However, the security factors are not considered in this research. In future, emerging paradigms of Blockchain, 5G, and Artificial Intelligence (AI) will be implemented with the proposed scheme. Also, real-time experiments will be conducted in further research.
