# Correction: COVID-19 prediction using Caviar Squirrel Jellyfish Search Optimization technique in fog-cloud based architecture

**DOI:** 10.1371/journal.pone.0303617

**Published:** 2024-05-09

**Authors:** Shanthi Amgothu, Srinivas Koppu

There is an error in the affiliation for authors Shanthi Amgothu and Srinivas Koppu. The correct affiliation is: School of Computer Science Engineering and Information Systems, Vellore Institute of Technology, Vellore, 632014, Tamil Nadu, India.
